# The Taxonomically Richest Liverwort Hemiboreal Flora in Eurasia Is in the South Kurils

**DOI:** 10.3390/plants11172200

**Published:** 2022-08-25

**Authors:** Vadim A. Bakalin, Ksenia G. Klimova, Daniil A. Bakalin, Seung Se Choi

**Affiliations:** 1 Laboratory of Cryptogamic Biota, Botanical Garden-Institute FEB RAS, Makovskogo Street 142, 690024 Vladivostok, Russia; 2 AXiiO Oy Company, Helsinki XR Center, Hämeentie, 135 A, 00560 Helsinki, Finland; 3 Team of National Ecosystem Survey, National Institute of Ecology, Seocheon 33657, Korea

**Keywords:** liverworts, diversity, distribution patterns, East Asia, Pacific, hemiboreal zone

## Abstract

The long coexistence of various floral elements, landscape diversity, and island isolation led to the formation of the richest Eurasian hemiboreal liverwort flora in the southern Kurils. This land that covers less than 5000 square kilometres and houses 242 species and two varieties of liverworts and hornworts. The flora ‘core’ is represented by hemiboreal East Asian and boreal circumpolar taxa. Other elements that have noticeable input in the flora formation are cool-temperate East Asian hypoarctomontane circumpolar and arctomontane. The distribution of some species is restricted to the thermal pools near active or dormant volcanoes or volcanic ash deposits; such species generally provide specificity to the flora. Despite the territorial proximity, the climate of each considered island is characterized by features that, in the vast majority of cases, distinguish it from the climate of the neighbouring island. The last circumstance may inspire the difference in the liverwort taxonomic composition of each of the islands. The comparison of the taxonomic composition of district floras in the Amphi-Pacific hemiarctic, boreal, and cool-temperate Asia revealed four main focal centres: East Kamchatka Peninsula and Sakhalin Island, the southern Sikhote-Alin and the East Manchurian Mountains, the mountains of the southern part of the Korean Peninsula, and the South Kurils plus northern Hokkaido. The remaining floras involved in the comparison occupy an intermediate position between these four centres.

## 1. Introduction

The South Kurils include Iturup, Kunashir, Shikotan, and a group of small Habomai islands. All are located in the southern part of the Kuril Island Chain to the north of Hokkaido and cover a small area of approximately 5000 square kilometres. Despite a small size, the South Kurils have unique vascular plant flora in which arctomontane and cool-temperate elements are present [1]. The liverworts in this promising flora were only poorly studied until the beginning of the 21st century. By 2005, only 53 liverwort species were known to the South Kurils [2], and no hornwort species were reported. The related group, mosses, was studied considerably more due to studies conducted in the late 1970s and early 1980s by moss specialists Bardunov and Cherdantseva [3]. Great advances in the knowledge of bryophytes in the South Kurils were made in the middle of the first decade of the 21st century, when approximately 4000 specimens of bryophytes were collected due to the joint efforts of a group of bryologists from 2005 to 2007. These collections have formed the background for the first reliable reports on the taxonomic diversity of these groups. For the South Kurils, as they are understood in this work (see Section 3), slightly over 200 species were reported [4], which is the great number for the district’s hemiboreal flora of relatively small-sized area.

Naturally, even at the time of publication for the checklist mentioned above [4], it could not be considered completely exhaustive since there were no data on taxonomic diversity in a number of areas on the islands. The incompleteness of the data presented in that paper (l.c.) has become more evident 13 years after publication. During the elapsed time, (1) new taxa have been described that did not appear in the previous checklist, (2) the taxonomic concept of a number of species was revised and sometimes drastically altered, (3) incorrect or doubtful species identifications were corrected, and (4) new materials were collected in a number of previously unstudied or insufficiently studied areas. It is worth mentioning that, in contrast to the materials on which the previous work was based [4], all newly collected materials were delivered alive to the cryptogamic biota laboratory (herbarium acronym VBGI), where oil bodies were studied and photographed for the vast majority of taxa. Several of the obtained photographs were published by Bakalin et al. [5]. The identity of several specimens was also tested using molecular genetic analysis. These facts suggest that the study of newly collected materials was conducted using a broader scale approach. As a result of the undertaken research, it is necessary to compile a new list of liverworts for the South Kurils. The publication of the new checklist along with a phytogeographic discussion is the main goal of the present study.

## 2. Results and Discussion

The main result of the field research and subsequent identification is an updated list of liverworts of the three southern Kuril Islands that includes 242 species and two varieties. These species are listed below. A total of 183 taxa are known for Iturup Island, 179 for Kunashir, and 148 for Shikotan. Thirty-two taxa are newly recorded for South Kurils, including four taxa (*Calypogeia japonica* Steph., *Calypogeia tosana* (Steph.) Steph., *Nipponolejeunea pilifera* (Steph.) S. Hatt., *Solenostoma hokkaidense* (Váňa) Váňa, Hentschel and Heinrichs). Although these were newly found in Iturup from field work in 2015 (Iturup Island), no localities were provided in the publication devoted to that island [6]. Below, we provide the list of the species recorded in the South Kurils with the data on the distribution within **I** (Iturup Island), **K** (Kunashir), and **S** (Shikotan). Doubtful records are listed together with confirmed records, although with corresponding references. Reidentified or, for other reasons, excluded species are listed in a separate section; this list includes 33 taxa. The field numbers are provided for the newly recorded taxa (the taxon known prior for the South Kurils in general is supplemented with field numbers only for the islands for which it is newly recorded), and the field number citation immediately follows the island name abbreviation (**I**, **K**, or **S**). No field numbers are cited for the species already known for the corresponding islands. The cited specimens from Iturup were collected by Bakalin (if otherwise not mentioned), whereas those from Kunashir and Shikotan were collected by Bakalin and Klimova. Some other changes (simply nomenclatural or resulting from the revisions of certain groups) are explained in the comments. If the species was reported for the South Kuril flora after 2009 but before the present account, the corresponding note and reference are provided. New records for islands are marked by asterisks following the island name abbreviation (in total 41 taxon were newly recorded on specific island), whereas new records for the South Kurils are indicated by an asterisk proceeding to the species name in the list. Simple name changes, e.g., all *Calycularia crispula* Mitt. Were transferred to *C. laxa* Lindb. and Arnell or, similarly, all *Bazzania ovifolia* (Steph.) S. Hatt. are now treated as *B. denudata* (Lindenb. et Gottsche) Trevis.) are not marked as newly recorded species, but are referenced with corresponding notices that the name used here is not an original name under which the species were previously reported in the flora. The homotypic synonyms used before are given in the checklist in the brackets. The simple transfer of species reports to the report under the heterotypic synonym of the same species and are not marked as new reports, although they are discussed in the species annotation.

The taxa are arranged alphabetically, and nomenclature follows Söderström et al. [7] with some updates from the recent literature (the most valuable is the ‘narrow’ genus concept in Solenostomataceae and acceptance of *Pseudolophozia* as distinct from *Barbilophozia*). The abbreviated island names where the taxon occurred are followed by the species name. The taxonomic or other notices (if any) are placed at the end of the comment sentence.

*Alobiellopsis parvifolia* (Steph.) R.M. Schust.—I.

*Anastrophyllum michauxii* (F. Weber) H. Buch—K.

*Aneura maxima* (Schiffn.) Steph.—I [newly recorded by Bakalin [8]], K* [K-50-10-20, K-50-14-20, K-55-11-20, K-47-4-20], S.

*Aneura pinguis* (L.) Dumort.—I, K, S.

*Anthelia *julacea* (L.) Dumort.—I [K-71-59-15, K-77-11-15, K-77-5-15, K-78-24-15, K-80-36-15, K-77-3-15, K-77-10-15, K-77-26-15, K-71-58-15, K-77-22-15, K-77-27a-15, K-80-37-15].

*Anthelia juratzkana* (Limpr.) Trev.—I, K, S.

*Barbilophozia barbata* (Schmid. ex Schreb.) Loeske—I.

*Barbilophozia hatcheri* (A. Evans) Loeske—I.

*Bazzania denudata* (Lindenb. et Gottsche) Trevis. (= *Bazzania ovifolia* (Steph.) S. Hatt.)—I, K, S; the synonymy with *B. ovifolia* is adopted after Bakalin [9].

*Bazzania tricrenata* (Wahlenb.) Lindb.—S; comment: reported for I, K, S by Bakalin et al. [4], but only the specimen from S is confirmed in subsequent works, others transferred to *B. denudata* [9].

*Bazzania trilobata* (L.) S. Gray—I, K, S.

*Blasia pusilla* L.—I, K, S.

*Blepharostoma minor* Horik.—K, S; comment: recorded for I, K, S, but genetically confirmed for K and S only, although likely occurs in I too [10].

*Blepharostoma neglectum Vilnet et Bakalin—I [K-32-15-07, K-71-32-15]; also cited in Bakalin et al. [10].

*Blepharostoma pseudominus Vilnet et Bakalin—K [K-42-1-06, K-44-21-18]; also cited in Bakalin et al. [10].

*Blepharostoma primum Vilnet et Bakalin—S [Shik-37-2-20, Shik-37-3-20, Shik-43-13-20, Shik-45-3-20, Shik-45-4-20, Shik-51-1-20, Shik-51-4-20, Shik-51-5-20, Shik-51-7-20].

*Calycularia laxa* Lindb. et Arnell—I, K, S; comment: these reports are also based on transfer of all *C. crispula* Mitt. from Russia to *C. laxa* following Konstnatinova and Mamontov [11].

*Calypogeia arguta* Nees and Mont.—I, K.

*Calypogeia integristipula* Steph.—I, K, S, doubtful report. It is likely that all specimens named as *C. integristipula* from the South Kurils should be transferred to recently described *C. pseudointegristipula* [12], but since we did not re-study the collection nor confirmed the transfers genetically, we retain the preceding name here.

*Calypogeia japonica Steph.—I [K-78-10-15].

*Calypogeia kamchatica*—I, K, S; based on transfer of previous reports of *C. neogaea* to the newly described species replaced *C. neogaea* in East Asia [12].

*Calypogeia muelleriana* (Schiffn.) Müll. Frib.—I, K, S.

*Calypogeia orientalis* Buczk. et Bakalin– I, K, S; as it was shown by Buczkovska et al. [13] that all reports of *C. azurea* from the Russian Far East should be transferred to *C. orientalis*.

*Calypogeia sphagnicola* (Arnell and J. Perss.) Warnst. et Loeske—I, S.

*Calypogeia subalpina* H. Inoue—I, K, S; comment: all these reports are based on the transfer of indications of *C. neesiana* (C. Massal. et Carestia) Müll. Frib. [12].

*Calypogeia suecica* (Arnell et J. Perss.) Müll. Frib.—I* [K-84-6-15, K-84-7-15], K, S.

*Calypogeia tosana (Steph.) Steph.—I [K-70-35-15].

*Cephalozia bicuspidata33* (L.) Dumort.—I, K, S.

*Cephalozia lacinulata* (J.B. Jack ex Gottsche et Rabenh.) Spruce—K.

*Cephalozia otaruensis* Steph.—I, K* [K-58-6-18, K-59-16-18, K-48-6-18, K-48-10-18, K-42-5-18, K-23-12-18, K-25-8-18, K-30-10-18, K-22-8-18], S.

*Cephaloziella arctogena* (R.M. Schust.) Konstant.—K.

*Cephaloziella divaricata* (Sm.) Schiffn.—I, K, S.

*Cephaloziella elachista* (J.B. Jack ex Gottsche et Rabenh.) Schiffn.—I, K.

*Cephaloziella uncinata* R.M. Schust.—I.

*Cheilolejeunea obtusifolia* (Steph.) S. Hatt.—I* [K-71-16-15, K-71-40-15, K-71-43-15, K-71-45-15, K-71-11-15, K-71-11-15], K* [K-43-2-18, K-30-9-18], S.

*Chiloscyphus rivularis* (Schrad.) Haszl.—I, K; comment: taxonomic status needs verification.

*Chiloscyphus fragilis* (A. Roth) Schiffn.—I, K; comment: taxonomic status needs verification.

*Chiloscyphus pallescens* (Ehrh. ex Hoffm.) Dumort.—I; comment: doubtful report by Blagodatskikh and Duda [14] that was probably arisen from wide treatment of the species and the specimens actually belong to *C. polyanthos* s.l.

*Chiloscyphus polyanthos* (L.) Corda—I, K, S.

*Cladopodiella fluitans* (Nees) H. Buch—I, K.

*Cladopodiella francisci* (Hook.) H. Buch ex Jørg.—I, K.

*Cololejeunea macounii* (Spruce) A. Evans—K, S* [K-38-8-20, K-41-5-20, Shik-33-6-20].

*Cololejeunea subkodamae* Mizut.—S.

*Conocephalum japonicum* (Thunb.) Grolle—I, K, S.

*Conocephalum salebrosum* Szweyk., Buczk. et Odrzyk.—I, K, S; comment: all reports of *C. conicum* (L.) Underw. were transferred here following Borovichev et al. [15].

*Cordaea flotoviana* Nees—I* [K-72-21-15], S; comment: reported by Mamontov et al. [16].

*Crossocalyx hellerianus* (Nees) Meyl.—I, K, S.

*Cryptocoleopsis imbricata* Amakawa—I, K.

*Diplophyllum albicans* (L.) Dumort.—I, K, S.

*Diplophyllum andrewsii* A. Evans—I, K, S.

*Diplophyllum taxifolium* (Wahlenb.) Dumort. var. *macrosticta* H. Buch—I, K, S; comment: var. *taxifolium* is not known in the South Kurils.

*Douinia plicata* (Lindb.) Konstant. et Vilnet (= *Macrodiplophyllum plicatum* (Lindb.) H. Perss.)—I, K, S.

*Endogemma caespiticia* (Lindenb.) Konstant., Vilnet et A.V.Troitsky (= *Solenostoma caespiticium* (Lindenb.) Steph.)—I, K.

*Eremonotus myriocarpus* (Carring.) Lindb. et Kaal.—I, K, S.

*Fossombronia alaskana* Steere & H. Inoue—I* [K-69-3-15], K, S* [K-62-2-20, K-47-2-20].

*Fossombronia japonica Schiffn.—S [K-43-1-20].

*Frullaniaappendiculata* Steph.—I* [K-69-7-15, K-71-58-15, K-71-14-15, K-71-15-15, K-71-9-15, K-71-12-15, K-71-4-15, K-71-5-15, K-71-6-15, K-71-2-15], K, S.

*Frullania austinii* J.J. Atwood, Vilnet, Mamontov et Konstant.—I* [K-83-4-15], K; comment: report for K is based on transfer of Asiatic specimens before named as *F. bolanderi* [4] to *F. austinii* (cf. [17].

*Frullania davurica* Hampe—I* [K-69-6-15, K-71-8-15, K-71-11-15, K-71-11-15, K-71-10-1], S.

*Frullania inflata* Gottsche—I, S; comment: these reports need to be revised in the view of current advances in the systematic of *Frullania* subsect. *Inflatae* [17].

*Frullania koponenii* S. Hatt.—K, S.

*Frullania muscicola* Steph.—I, K, S; comment: these reports need to be revised in the view of current advances in the systematic of *Frullania*.

*Frullania takayuensis Steph.—K [K-44-4-18, K-38-1-18], S [K-64-4-20, Shik-37-6-20, Shik-42-6-20, Shik-44-6-20].

*Frullania usamiensis* Steph.—K; comment: recorded from K as new for Russia by Mamontov et al. [18].

*Fuscocephaloziopsis catenulata subsp. *nipponica* (S.Hatt.) Váňa et L.Söderstr.—K [K-60-2a-18].

*Fuscocephaloziopsis* cf. *connivens* (Dicks.) Váňa et L.Söderstr. (= *Cephalozia connivens* (Dicks.) Lindb.)—K; comment: the cited specimen [4] was collected in Kunashir, but mistakenly indicated as Shikotan in the cited paper. It contains plants with characteristically large cells in the leaf, but the insertion and decurrency of the leaf is more similar to that in *F. pleniceps*. We are not sure plants in the specimen belong to ‘true’ *F. pleniceps* or *F. connivens*.

*Fuscocephaloziopsis leucantha* (Spruce) Váňa et L.Söderstr. (= *Cephalozia leucantha* Spruce)—I, K, S.

*Fuscocephaloziopsis loitlesbergeri (Schiffn.) Váňa et L.Söderstr.—K [K-57-7-18].

*Fuscocephaloziopsis lunulifolia* (Dumort.) Váňa et L.Söderstr. (= *Cephalozia lunulifolia* (Dumort.) Dumort.)—I, K, S.

*Fuscocephaloziopsis pachycaulis* (R.M.Schust.) Váňa et L.Söderstr. (= *Cephalozia pachycaulis* R.M. Schust.)—I, K.

*Fuscocephaloziopsis pleniceps* (Austin) Váňa et L.Söderstr. (= *Cephalozia pleniceps* (Austin) Lindb.)—I, K.

*Geocalyx graveolens* (Schrad.) Nees—I, K, S.

*Geocalyx lancistipulus* (Steph.) S. Hatt.—K; comment: the delimitation of *G. graveolens* and *G. lancistipulus* in sterile conditions (all known *Geocalyx* specimens from the Kurils are sterile) is doubtful. Taking into account the phytogeographic reasons, the only species possible in the South Kurils should be *G. lancistipulus*.

*Gymnocolea inflata* (Huds.) Dumort.—I, K, S* [Shik-43-15-20, Shik-43-16-20].

*Gymnocolea marginata* (Steph.) S. Hatt.—I, K; comment: the taxonomic status is unclear, and we are not inclined to treat it as conspecific with *G. inflata*, but more robust confirmations including DNA sets analysis are required in this case.

*Gymnomitrion adustum* Nees (= *Marsupella adusta* (Nees) Spruce)—K.

*Gymnomitrion concinnatum* (Lightf.) Corda—I, K; comment: the species was recorded for I, K, S [4], but is confirmed for I, K only, whereas specimens from S belong to *G. faurianum* [19].

*Gymnomitrion faurieanum* (Steph.) Horik.—I, K, S; comment: newly reported for all cited islands by Bakalin [19].

*Gymnomitrion parvitextum* (Steph.) Mamontov, Konstant. et Potemkin—I, K* [K-48-17-18], S [K-53-18-20]; comment: the taxon was indicated for I as *G. commutatum* in [4] due to wider treatment of the latter taxon than now adopted.

*Haplomitrium hookeri* (Sm.) Nees—I.

*Harpanthus flotovianus* (Nees) Nees—I, K, S.

*Harpanthus scutatus (F. Weber et D. Mohr) Spruce—S [Shik-37-1-20].

*Hattorianthus erimonus* (Steph.) R.M. Schust. et H. Inoue—I* [K-71-53-15, K-72-1-15, K-71-35-15, K-70-55-15, K-80-15-15, K-80-16-15, K-80-11-15], S.

*Herbertus aduncus* (Dicks.) Gray—K* [K-31-11-18, K-31-14-18], S; comment: the relationships between *H. dicranus* and *H. aduncus* need clarification. As Juslén [20] has mentioned, the type of *H. aduncus* contains the only one plant of *H. aduncus* whereas other plants supposedly belong to *H. dicranus*. If two shoots in the specimen are really different, the evidence as to why this is not a simple morphological variation within one species should be provided.

*Herbertus buchii* Juslen—S; comment: the species described by Juslén [20] seems to be based on simply depauperate plants of *H. dicranus*, but this was never tested by genetic methods and now we avoid the formal synonymyzation of these two names.

*Herbertus dicranus (Taylor ex Gottsche, Lindenb. et Nees) Trevis.—S [K-39-10-20, K-39-13-20, K-41-3-20, Shik-40-1-20].

*Heterogemma laxa (Lindb.) Konstant. et Vilnet—K [K-57-8-18, K-57-18-18].

*Hygrobiella intermedia* Bakalin et Vilnet—I, S; comment: newly recorded for those islands by Bakalin and Vilnet [21].

*Hygrobiella laxifolia* (Hook.) Spruce—I [K-75-8-15]; comment: it was not confirmed for the flora based on then existing specimens in Bakalin and Vilnet [21], but in 2015, it was collected in Iturup from where it was recorded.

*Hygrobiella squamosa* Bakalin et Vilnet –I, K; comment: newly recorded by Bakalin and Vilnet [21].

*Isopaches bicrenatus* (Schmid. ex Hoffm.) H. Buch—I, K.

*Jungermannia atrovirens Dumort.—I [K-80-13-15, K-80-2-15, K-80-23-15, K-80-8-15, K-71-49-15], K [K-23-8-18].

*Jungermannia borealis Damsh. et Vana—I [K-82-5-15, K-82-1-15].

*Jungermannia eucordifolia* Schljakov—I, K, S.

*Jungermannia exsertifolia* Steph.—I, K, S.

*Jungermannia pumila* With.—I, K, S; comment: this small paroicous *Jungermannia* formally referred to *J. pumila* may represent another as yet described species, since it possesses biconcentric oil bodies not known in European populations of the species.

*Kurzia makinoana* (Steph.) Grolle—I, K, S.

*Lejeunea flava (Sw.) Nees—S [K-45-4a-20, K-45-5-20, Shik-37-15-20, Shik-37-28-20].

*Lejeunea japonica* Mitt.—K* [K-30-6-18, K-30-9a-18], S.

*Lejeunea otiana* S. Hatt.—S.

*Lepidozia reptans* (L.) Dumort.—I, K, S.

*Liochlaena subulata* (A. Evans) Schljakov—I, K, S.

*Lophocolea cuspidata* (Nees) Limpr.—I, K, S.

*Lophocolea heterophylla* (Schrad.) Dumort.—I, K, S.

*Lophocolea itoana* H. Inoue—I; comment: doubtful record, material was studied without oil bodies (those should be biconcentric in this species, in other traits the species is highly similar to *L. cuspidata*). Moreover, there are no specimens from the Russian Far East that would be studied in fresh conditions with oil bodies available for study; the occurrence of the taxon in Russia as well as the taxonomic status itself may be questioned.

*Lophocolea minor* Nees—I, K, S.

*Lophozia guttulata* (Lindb. et Arnell) A. Evans—I, K, S.

*Lophozia lacerata* N. Kitag.—I; comment: the taxonomic status of the taxon is questionable.

*Lophozia lantratoviae Bakalin—I [K-76-25-15].

*Lophozia obscura* (Bakalin) A.V. Troitsky, Bakalin et Fedosov—I; comment: it was described as new species under *Schistochilopsis obscura* Bakalin in [22] from this island.

*Lophozia savicziae* Schljakov—I, S* [K-53-14-20].

*Lophozia schusteriana Schljakov—K [K-34-30-18]; comment: the species status of the taxon and the identity of East Asian populations with those from Ellesmere Island (the type locality for the taxon) need confirmation.

*Lophozia silvicola* H. Buch—I, K, S.

*Lophozia silvicoloides* N. Kitag.—I, K, S.

*Lophozia ventricosa6* (Dicks.) Dumort.—I, K.

*Lophozia wenzelii* (Nees) Steph.—K; comment: the report needs molecular verification.

*Marchantia alpestris* (Nees) Burgeff—I, K.

*Marchantia latifolia* Gray—I, K* [K-35-21-18], S.

*Marchantia paleacea* Bertol.—K.

*Marchantia polymorpha* L. (sensu *M. aquatica* (Nees) Burgeff)—K.

*Marsupella alata* S. Hatt.—I, K* [K-48-4-18, K-35-11-18], S.

*Marsupella *apertifolia* Steph.—I [K-77-6-15, K-77-7-15, K-79-12-15, K-79-40-15, K-79-19-15, K-79-26-15, K-79-31-15, K-79-2-15, K-79-2-15, K-77-31a-15], K [K-31-4-18].

*Marsupella apiculata* Schiffn. (= *Gymnomitrion apiculatum* (Schiffn.) Müll. Frib.)—I, K.

*Marsupella boeckii* (Aust.) Lindb. ex Kaal.—K, S.

*Marsupella condensata (Aongstr. ex C. Hartm.) Lindb. ex Kaal.—I [K-76-26-15].

*Marsupella disticha* Steph.—I [K-78-9-15, K-75-10-15].

*Marsupella funckii* (F.Weber et D.Mohr) Dumort.—K.

*Marsupella pseudofunckii* S. Hatt.—I, K* [K-30-8-18, K-31-16-18].

*Marsupella sprucei* (Limpr.) H. Bernet—I.

*Marsupella tubulosa* Steph.—I, K, S; comment: all reports of ‘*M. sphacelata*’ by Bakalin et al. [4] belong here.

*Mesoptychia heterocolpos* (Thed. ex Hartm.) L.Söderstr. et Váňa (= *Leiocolea heterocolpos* (Thed. ex Hartm.) H. Buch var. *heterocolpos*)—I.

*Mesoptychia rutheana* (Limpr.) L.Söderstr. et Váňa—I, leg. Borovichev, the mire in the foot of Kudryavyi Volcano (KPABG).

*Metacalypogeia cordifolia* (Steph.) H. Inoue—I* [K-71-19-15], K, S.

*Metasolenostoma ochotense* (Bakalin et Vilnet) Vilnet et Bakalin—I, K, S; comment: reported by Bakalin et al. [23].

*Metasolenostoma orientale* Bakalin et Vilnet—I, K; comment: reported by Bakalin et al. [23].

*Metzgeria lindbergii* Schiffn.—I, K, S.

*Metzgeria pubescens* (Schrank) Raddi (= *Apometzgeria pubescens* (Schrank) Kuwah.)—K, S.

*Metzgeria temperata* Kuwah.—K, S.

*Microlejeunea punctiformis* (Taylor) Steph.—K, S; comment: the difference of this species from *Lejeunea ulicina* (Taylor) Gottsche et al. (under that it was reported for South Kurils in [4]) was argued by Zhu and So [24]. Our specimens belong to *Microlejeunea punctiformis*.

*Moerckia blyttii* (Moerck ex Hornem.) Brockm.—S; comment: doubtful report, the cited specimen (cf. [4]), K-54-3-07, was not found in the present study, but another specimen not cited in [4] was later identified by Mamontov et al. [16] as *Cordaea flotoviana* Nees. However, from the phytogeographical point of view, the presence of *M. blyttii* in S is quite possible.

*Mylia anomala* (Hook.) S.Gray—I, K, S.

*Mylia taylorii* (Hook.) S. Gray—I, K, S.

*Mylia verrucosa* Lindb.—I, K, S.

*Nardia assamica* (Mitt.) Amakawa—I, K, S.

*Nardia breidleri* (Limpr.) Lindb.—I, K* [K-46-2-18, K-47-1-18, K-47-6-18, K-47-12-18, K-47-23-18, K-47-27-18].

*Nardia compressa* (Hook.) Gray—I, K.

*Nardia geoscyphus* (De Not.) Lindb.—I.

*Nardia geoscyphus* (De Not.) Lindb. var. *dioica* Bakalin—S.

*Nardia harae* Amakawa—I, K, S; comment: the status of the taxon is unclear; in minor details, it differs from *N. scalaris* distributed in North-East Europe and North America, and we estimate *N. harae* to be distinct from *N. scalaris* s. str. At the species level, the present report also includes all previous records of *N. scalaris* for the South Kurils; ‘true’ *N. scalaris* presumable does not occur in Pacific Asia, although this suggestion needs confirmation.

*Nardia hiroshii* Amakawa—I, K* [K-47-18-18].

*Nardia insecta Lindb.—S [K-55-4-20, K-57-13-20, Shik-50-3-20].

*Nardia japonica* Steph.—I [K-76-24-15, K-76-8-15], K?, S [Shik-32-5a-20]; comment: the species was recorded [4] based on study of previously dried material without oil bodies available for study and prior to the description of *Nardia pacifica* (described by Bakalin and Klimova [25]). Some of these reports surely belong to *N. pacifica*. *Nardia japonica* was confirmed based on fresh material comparison for Iturup and Shikotan islands only (the studied specimens are provided here).

*Nardia pacifica* Bakalin—I; comment: reported for Iturup in [25] based on study of fresh material with oil bodies available. Some of earlier reports of *N. japonica* may belong to this species.

*Nardia subclavata* (Steph.) Amakawa—I, K, S.

*Nardia unispiralis* Amakawa—I.

*Neohattoria herzogii* (Hatt.) Kamim.—I, K, S.

*Neoorthocaulis attenuatus* (Mart.) L.Söderstr., De Roo et Hedd. (= *Orthocaulis attenuatus* (Mart.) A. Evans)—I, K* [K-47-24-18].

*Nipponolejeunea pilifera (Steph.) S. Hatt.—I [K-69-15-15, K-69-8-15, K-69-9-15, K-71-10-15, K-71-12-15, K-71-14-15, K-71-15-15, K-71-3-15, K-71-4-15, K-71-5-15, K-71-6-15, K-71-7-15, K-71-9-15]; comment: this species is newly recorded for Russia and this record was briefly discussed by Bakalin et al. [6], but no specimen citation were provided in l.c.

*Nipponolejeunea subalpina* (Horikawa) S. Hatt.—I, K, S.

*Nowellia curvifolia* (Dicks.) Mitt.—K.

*Obtusifolium obtusum* (Lindb.) S.W. Arnell—K; comment: this is a doubtful report, the vouchers were not found in the course of the present study.

*Odontoschisma denudatum* (Mart.) Dumort.—K, S.

*Odontoschisma elongatum (Lindb.) Evans—I [K-70-9-15, K-70-17-15, K-77-3-15].

*Odontoschisma macounii* (Austin) Underw.—K.

*Odontoschisma pseudogrosseverrucosum Gradst., S.C. Aranda et Vanderp.—K [K-30-12-18].

*Pallavicinia levieri Schiffn.—I [K-70-53-15].

*Pallavicinia subciliata* (Austin) Steph.—K; comment: the earlier report of *P. lyellii* [4] also belong to this species [16].

*Pedinophyllum truncatum* (Steph.) H. Inoue—I, K, S.

*Pellia endiviifolia* (Dicks.) Dumort.—I, K, S.

*Pellia epiphylla* (L.) Corda—I.

*Pellia neesiana* (Gottsche) Limpr.—I, K, S.

*Phaeoceros carolinianus (Michx.) Prosk.—K [K-38-9-18, K-38-10-18], S [K-42-2-20, Shik-39-1-20].

*Plagiochila hakkodensis Steph.—S [K-39-18-20, Shik-37-2-20, Shik-40-23-20, Shik-53-21-20, Shik-53-6-20].

*Plagiochila ovalifolia* Mitt.—I, K, S.

*Plagiochila porelloides* (Torrey ex Nees) Lindenb.—I, K, S.

*Plagiochila satoi S. Hatt.—I [K-80-26-15, K-73-6-15], K [K-37-11-18], S [K-59-11-20, Shik-51-10-20]; comment: the status of the taxon is doubtful due to unclear relationships with *P. porelloides* (both treated by So [26] as conspecific); we, however, suggest two taxa may be distinguished at the species level.

*Plectocolea hattoriana* Amakawa—I* [K-78-13-15, K-78-35-15, K-78-38-15, K-78-37a-15, K-78-25-15], K* [K-43-12-18, K-43-16-18, K-43-17-18, K-34-47-18, K-34-1-18, K-23-3-18], S—confirmed.

*Plectocolea horikowana Amakawa—I [K-80-30-15, K-80-33-15, K-80-28-15, K-80-28a-15], S [K-55-1-20, K-55-6-20].

*Plectocolea infusca* Mitt. var. *infusca*—I, K, S; comment: the distribution is also confirmed by Bakalin [27].

*Plectocolea infusca* var. *recondita* Bakalin—I, K, S (data by Bakalin [27]).

*Plectocolea kurilensis* (Bakalin) Bakalin et Vilnet (= *Plectocolea flagellata* S. Hatt. var. *kurilensis* Bakalin)—I, K, S; comment: the distribution is confirmed by Bakalin [27].

*Plectocolea ovalifolia* (Amakawa) Bakalin et Vilnet (= *Plectocolea infusca* Mitt. var. *ovalifolia* Amakawa)—I, K, S; comment: the distribution is confirmed by Bakalin [27].

*Plectocolea rigidula* S. Hatt.—K, S; comment: the distribution is confirmed by Bakalin [27].

*Plectocolea vulcanicola* Schiffn.—I, K, S; comment: the distribution is confirmed by Bakalin [27].

*Pleurocladula albescens* (Hook.) Grolle—I, K.

*Porella fauriei* (Steph.) S. Hatt.—I, K, S.

*Porella grandiloba* Lindb.—I* [K-73-7-15, K-73-11-15, K-73-11a-15, K-73-17-15], K.

*Preissia quadrata* (Scop.) Nees—I, K, S.

*Pseudolophozia debiliformis* (R.M. Schust. et Damsh.) Konstant. and Vilnet (= *Protolophozia debiliformis* (Schust.) Konstant.)—I, K* [K-47-7-18], S.

*Pseudolophozia sudetica* (Nees ex Huebener) Konstant. et Vilnet (= *Lophozia sudetica* (Nees ex Huebener) Grolle)—I.

*Ptilidium californicum* (Aust.) Pears.—I.

*Ptilidium ciliare* (L.) Hampe—I, S.

*Ptilidium pulcherrimum* (Weber) Vain.—I, K, S.

*Radula brunnea* Steph.—S; comment: the species was treated in details by Bakalin and Klimova [28].

*Radula complanata* (L.) Dumort.—I, K, S; comment: the distribution is also confirmed by Bakalin and Klimova [28].

*Radula constricta* Steph.—I, K, S; comment: the distribution is also confirmed by Bakalin and Klimova [28].

*Radula fauriana* Steph.—I [K-48-4-05, K-70-39-15], comment: newly reported for Kurils by Bakalin and Klimova [28], but no vouchers provided in l.c.

*Radula japonica* Gottsche—I, K, S; comment: the distribution is also confirmed by Bakalin and Klimova [28].

*Radula obtusiloba* Steph.—I, K, S; comment: the distribution is also confirmed by Bakalin and Klimova [28].

*Reboulia hemisphaerica* (L.) Raddi subsp. *orientalis* R. M. Schust.—I* [K-80-25-15, K-82-6-15], K, S.

*Riccardia aeruginosa* Furuki—I, K; comment: also confirmed by Bakalin [8].

*Riccardia chamedryfolia* (With.) Grolle—I, K, S* [Shik-43-2-20].

*Riccardia decrescens* (Steph.) S. Hatt.—I, K, S; comment: also confirmed by Bakalin [8]. All earlier reports of *R. multifida* are placed here.

*Riccardia latifrons* (Lindb.) Lindb.—K, S* [K-48-6-20, Shik-42-12-20, Shik-44-10-20]; comment: confirmed for K by Bakalin [8].

*Riccardia palmata* (Hedw.) Carruth.—I* [K-84-3-15], K, S.

*Riccardia subalpina* Furuki—I, K* [K-34-33-18, K-34-36-18]; comment: the vouchers from Iturup Island were not found in the course of the revision [8] but later the species was newly found in Kunashir.

*Riccardia vitrea* Furuki—K.

*Riccia fluitans* Lindenb.—I; comment: originally [4] the taxon was recorded for S. Later Borovichev and Bakalin [29] showed the voucher contains *R. huebeneriana* and therefore excluded the taxon from Shikotan liverwort list. However, in the same paper, they reported *R. fluitans* for Iturup Island and thus the liverwort flora of South Kurils still contain this species within.

*Riccia huebeneriana* Lindenb.—S; comment: newly reported by Borovichev and Bakalin [29] based on original erroneous report of *R. fluitans* for S.

*Scapania apiculata* Spruce—K.

*Scapania crassiretis* Bryhn—I.

*Scapania curta* (Mart.) Dumort.—I; comment: the distribution is confirmed by Choi et al. [30].

*Scapania diplophylloides* Amakawa et S. Hatt.—I, K, S; comment: the distribution is confirmed by Choi et al. [30].

*Scapania gigantea* Horik.—K, S; comment: newly recorded by Potemkin et al. [31].

*Scapania hirosakiensis* Steph.—I, K, S; comment: the distribution is confirmed by Choi et al. [30].

*Scapania irrigua* (Nees) Nees—I* [K-78-1-15, K-83-2-15], K.

*Scapania lingulata* H. Buch—I, K; comment: newly recorded for South Kurils by Choi et al. [30].

*Scapania mucronata H. Buch—S [Shik-35-12-20].

*Scapania paludicola* Loeske et Müll. Frib.—I, K, S.

*Scapania paludosa* (Müll. Frib.) Müll. Frib.—I, K, S; comment: the distribution is confirmed by Choi et al. [30], but specimens should be restudied in the light of newly described *S. pseudouliginosa* to which some of previous reports of this species may belong [31].

*Scapania parvidens* Steph.—I, K, S; comment: newly reported by Choi et al. [30].

*Scapania parvifolia* Warnst.—I* [K-71-39a-15, K-73-14-15, K-71-39-15], K, S* [Shik-35-10-20, Shik-51-10-20, Shik-51-11-20, Shik-51-8-20, Shik-51-8a-20].

*Scapania parvitexta* Steph.—I, K, S.

*Scapania scandica* (Arnell et H. Buch) Macvicar—S; comment: doubtful report, provided by Bakalin et al. [4] for S basing on the collections identified by Nyushko (coauthor of the cited paper, but none of vouchers were found in subsequent revisions and possibly all reports are based on *S. parvifolia* before sometimes treated as the form within *S. scandica*.

*Scapania subalpina* (Nees ex Lindenb.) Dumort.—I, K; comment: the distribution is confirmed by Choi et al. [30].

*Scapania umbrosa* (Schrad.) Dumort.—K; comment: the distribution is confirmed by Choi et al. [30].

*Scapania undulata* (L.) Dumort.—I, K, S; comment: the distribution is confirmed by Choi et al. [30].

*Schistochilopsis cornuta* (Steph.) Konstant.—I, K, S.

*Schistochilopsis incisa* (Schrad.) Konstant.—I, K, S.

*Schistochilopsis pacifica* Bakalin—I; comment: paratype of the species is from I [22].

*Solenostoma bilobum* (S.Hatt. ex Amakawa) Potemkin et Nyushko (= *Plectocolea biloba* Amakawa)—K* [K-31-6-18, K-31-20-18], S.

*Solenostoma emarginatum (Amak.) Váňa, Hentschel et J. Heinrichs—K [K-31-3-18, K-31-21-18].

*Solenostoma hokkaidense (Váňa) Váňa, Hentschel et J. Heinrichs—I [K-78-28-15, K-75-8a-15, K-75-8-15], K [K-48-11-18, K-42-10-18, K-35-2-18], S [K-57-6-20, Shik-48-16-20].

*Solenostoma hyalinum* (Lyell) Mitt. (= *Plectocolea hyalina* (Lyell) Mitt.)—I, K, S; comment: the status of the specimens from South Kurils need clarification, probably they belong to the other not yet described taxon.

*Solenostoma pseudopyriflorum* Bakalin et Vilnet—I, K* [K-51-2a-18, K-31-8-18], S.

*Solenostoma rossicum* Bakalin et Vilnet—I, K, S; comment: newly recorded by Bakalin [27].

*Solenostoma rotundatum* Amakawa—K* [K-54-1-18, K-38-22-18].

*Solenostoma subellipticum* (Lindb. ex Heeg) R.M. Schust.—I; comment: based on transfer of original *Plectocolea otiana* and *P. harana* reports by Bakalin [27].

*Sphenolobus minutus* (Schreb.) Berggr.—I, K, S* [Shik-54-10-20, Shik-54-14-20].

*Syzygiella autumnalis* (DC.) K.Feldberg, Váňa, Hentschel et Heinrichs (= *Crossogyna autumnalis* (DC) Schljakov)—I, K, S.

*Trichocolea tomentella* (Ehrh.) Dumort.—K, S; comment: the identity of Far Eastern populations of the species and European ones (where type locality of *T. tomentella*) may be questioned and this problem requires further investigations.

*Trilophozia quinquedentata* (Huds.) Bakalin (= *Tritomaria quinquedentata* (Huds.) H. Buch)—I, K, S.

*Tritomaria exsecta* (Schmid. ex Schrad.) Loeske—I, K, S.

*Wiesnerella denudata* (Mitt.) Steph.—I, S; comment: recorded by Borovichev and Bakalin [32].

### Excluded Taxa (In Comparison with the List Provided by Bakalin et al., 2009)

*Bazzania bidentula* (Steph.) Steph.—excluded from the Russian flora, the specimen from Iturup (K-56-17-05) is transferred to *B. parabidentula* [9].

*Bazzania japonica* (Sande Lac.) Lindb.—excluded from the Russian flora, the specimens cited for Kurils were transferred to *B. denudata* [9].

*Bazzania ovifolia* (Steph.) S. Hatt.—the synonymy with *B. denudata* is confirmed by Bakalin [9] and all reports are transferred to the latter.

*Blepharostoma trichophyllum* (L.) Dumort. var. *trichophyllum*—excluded from the flora [10] as based on misidentifications for other newly described taxa.

*Calycularia crispula* Mitt.—excluded from the Russian flora, all records transferred to *C. laxa* [11].

*Calypogeia azurea* Stotler et Crotz—excluded from the flora of the Russian Far East, all records transferred to *C. orientalis* [13].

*Conocephalum conicum* (L.) Underw.—excluded the flora of the Russian Far East, all specimens transferred to *C. salebrosum* [15].

*Frullania bolanderi* Austin—excluded, all reports from the Russian Asia presumable belong to the recently described *F. austinii* [17].

*Gymnomitrion alpinum* (Gottsche ex Husn.) Schiffn. (under *Marsupella alpina* (Gott. Ex Limpr.) H. Bernet)—excluded from the flora of the South Kurils, specimens belong to various taxa, and some of them should be described as new.

*Gymnomitrion commutatum* (Limpr.) Schiffn. (under *Marsupella commutata* (Limpr.) H. Bern.)—excluded from the flora of the South Kurils, all reports belong to *G. parvitextum*.

*Iwatsukia jishibae* (Steph.) N. Kitag.—excluded from the flora of the South Kurils as misidentification of the modification of *Fuscocephaloziopsis leucantha* with sporadic underleaves.

*Lejeunea cavifolia* (Ehrh.) Lindb.—excluded from the flora of the Russian Far East, plants belong to *L. japonica* [33].

*Lejeunea ulicina* (Taylor) Gottsche, Lindenb. et Nees —excluded from the flora of the South Kurils due to transfer of all specimens to *Microlejeunea punctiformis*—morphologically similar, but nevertheless different species, as it was argued by Zhu and So [24].

*Marsupella sphacelata* (Gieseke ex Lindenb.) Dumort.—excluded from the flora of South Kurils, all reports belong to *M. tubulosa*.

*Nardia scalaris* S. Gray subsp. *scalaris*—excluded, all previously cited specimens belong to *Nardia harae*, a taxon of somewhat questionable status.

*Pallavicinia lyellii* (Hook.) Carruthers—excluded, the report is misidentification of *P. subciliata* [16].

*Pedinophyllum interruptum* (Nees) Lindb.—excluded from the flora of South Kurils, cited specimens belong to *P. truncatum*.

*Plectocolea harana* Amakawa—excluded, cited specimens from Iturup were re-identified as *Solenostoma subellipticum* by Bakalin [27].

*Plectocolea infusca* Mitt. var. *ovicalyx* (Steph.) Bakalin—reduced to the synonym of *P. infusca* var. *infusca* [34]. However, some specimens before identified as var. *ovicalyx* belong to recently described *P. infusca* var. *recondita* [27].

*Plectocolea otiana* S. Hatt.—excluded from the flora of the South Kurils, specimens transferred to *Solenostoma subellipticum* by Bakalin [27].

*Plectocolea rupicola* (Amakawa) Bakalin.—excluded, specimens re-identified as *P. kurilensis* [27].

*Plectocolea virgata* Mitt.—excluded from the Russian flora, specimen belong to *Solenostoma rossicum* [27].

*Riccardia multifida* (L.) Gray—excluded, following Bakalin [8], all reports of this species in the Russian Far East belong to *R. decrescens*.

*Scapania ampliata* Steph.—excluded from the Russian flora by Choi et al. [30].

*Scapania integerrima* Steph.—excluded from the Russian flora by Choi et al. [30].

*Scapania ligulata* Steph.—excluded as misidentification of *S. parvidens* by Choi et al. [30].

*Scapania uliginosa* (Lindenb.) Dumort.—excluded from the flora of South Kurils by Choi et al. [30]. The report in Hepaticae Rossicae Exsiccatae (#300) for Iturup belongs to *S. gigantea* (Potemkin, pers. comm.).

*Schistochilopsis opacifolia* (Culm. ex Meyl.) Konstant.—excluded due to confirmed synonymy with *S. incisa* by Bakalin et al. [35].

*Solenostoma fusiforme* (Steph.) R.M. Schust.—excluded from the Russian flora, all reports transferred to *Metasolenostoma orientale* [27].

*Solenostoma koreanum* Steph.—excluded from the Russian flora, all reports transferred to *Metasolenostoma ochotense* [27].

*Solenostoma jenseniana* (Grolle) Bakalin (= *Solenostoma pusillum* (C.E.O. Jensen) Steph.)—excluded by Bakalin [27] from the flora of South Kuril flora as misidentification of *S. rossicum*.

*Solenostoma rishiriense* Amakawa—excluded from the Russian flora, reports are misidentifications of various taxa [27].

*Solenostoma sphaerocarpum* (Hook.) Steph.—excluded as based on misidentification of *S. rossicum*.

The total known liverwort and hornwort diversity of the South Kurils includes 242 species and two varieties. The ‘core’ of the flora is formed by 104 species known on all three islands. The vast majority of the flora ‘core’ taxa possess a generally boreal circumpolar distribution far beyond the East Asian floristic region. In addition, the sizable proportion in these ‘core’ taxa (17 species) is characterized by East Asian distribution: *Cheilolejeunea obtusifolia*, *Conocephalum japonicum*, *Frullania appendiculata*, *Gymnomitrion faurianum*, *Marsupella alata*, *Metacalypogeia cordifolia*, *Mylia verrucosa*, *Plectocolea kurilensis*, *Radula japonica*, *Radula obtusiloba*, *Scapania diplophylloides*, *Scapania hirosakiensis*, *Scapania parvidens*, *Scapania parvitexta*, *Schistochilopsis cornuta*, *Solenostoma hokkaidense*, and *Solenostoma pseudopyriflorum*.

Within the three studied islands, there were three island pairs, and a certain number of common taxa characterized each pair. Only in Iturup and Kunashir were 33 species found; in Iturup and Shikotan, 9 species; and in Kunashir and Shikotan, 17 species.

Common species in Iturup and Kunashir have more northern distribution patterns than could be expected from the phytogeographic position of both islands in the hemiboreal zone, although easily predictable due to their large (much larger than in Shikotan) variation in elevation above sea level. Many of them have boreal to arctic-boreal distribution, such as *Endogemma caespiticium*, *Cladopodiella* spp., *Frullania austinii*, *Fuscocephaloziopsis pachycaulis*, *F. pleniceps*, etc. In addition, a special case is the distribution on both islands of *Cryptocoleopsis imbricata*, a species of young volcanogenic substrate (scoria and volcanic ash mainly that in the crevices of tufa and other pyroclastic-derived cliffs). Iturup and Shikotan common species mainly possess boreal and Arctic-boreal circumpolar distributions, with the exception of two bright East Asian taxa: *Hattorianthus erimonus* and *Wiesnerella denudata*. All these species may also be found on Kunashir Island. Kunashir and Shikotan common taxa mostly have East Asian (temperate to arctic-alpine) or widely temperate distributions as determined from their southern location in comparison with Iturup Island: *Blepharostoma minus*, *Cololejeunea macounii*, *Frullania koponenii*, *Frullania takayuensis*, *Lejeunea japonica*, *Metzgeria temperata*, *Microlejeunea punctiformis*, *Phaeoceros carolinianus*, *Plectocolea rigidula*, *Scapania rigidula*, *Solenostoma bilobum*, and *Trichocolea tomentella*. Other floral elements within the common species fraction (boreal, arctomontane, hemiboreal circumpolar, etc.) are in the minority and include only five species.

The specificity of the certain island is quite large: The distribution of 37 species is restricted to Iturup, 25 to Kunashir, and 17 to Shikotan.

The species specific to Iturup form quite a heterogeneous group, including East Asian (*Alobiellopsis parvifolia*, *Calypogeia japonica*, *C. tosana*, *Lophocolea itoana*, *Marsupella apertifolia*), arctomontane (*Anthelia julacea*, *Cephaloziella uncinata*), and even hemiboreal Asian (*Lophozia lantratoviae*) taxa. The taxa specific to Kunashir also form a heterogeneous entity, in which East Asian temperate taxa are more numerous than taxa with other distribution types. Examples are *Fuscocephaloziopsis catenulata* subsp. *nipponica*, *Odontoschisma pseudogrosseverrucosum*, *Pallavicinia subciliata*, *Riccardia vitrea*, and *Solenostoma emarginatum*. The species specific to Shikotan include both predominantly temperate East Asian taxa (*Cololejeunea subkodamae*, *Fossombronia japonica*, *Lejeunea otiana*, *Plagiochila hakkodensis*) and circumboreal taxa, which theoretically can be found on other islands in the South Kurils.

As mentioned above, the total known taxonomic liverwort diversity in the South Kurils includes 242 species, which is the highest number for hemiboreal flora of similar and even much larger sizes. If to directly compare the number of known species in the South Kurils with the species numbers for European countries [36], then very few countries would be found ahead of the South Kurils in terms of the number of species, although such a comparison is certainly not appropriate on the basis of logic. Moreover, the highest numbers exceeded South Kurils flora by no more than 30%. Among the countries with similar taxonomic diversity to the South Kurils are the following (despite the significant differences in the area, the knowledge history, and landscape diversity): Austria—260 species, Switzerland—263, Germany—252, Finland—222, Ireland—235, Poland—233, Sweden—262, and Slovakia—223 species. The South Kurils are far ahead of floristically rich and well-studied countries such as Belgium (192 species) and Denmark (144 species), and somewhat lag behind the most taxonomically rich countries: Great Britain (293 species), Spain (276), France (308), and Norway (277).

Of course, such a comparison is surely inappropriate from the size of the compared units but clearly illustrates the taxonomic richness of the liverwort flora of the South Kurils. There are few data to compare the diversity in the South Kurils with adjacent areas. The large area (approximately 200,000 square km) lying mostly in the temperate zone of the Korean Peninsula houses 326 liverwort and hornwort species [37]. However, if we compare the number of species with that known for Hokkaido Island, which is much larger in area (83,424 square km), more diverse in terms of landscape and climate, and lies mostly in the temperate zone, then the comparison will be in favour of the South Kurils: 182 species are known in Hokkaido [38] versus 242 in the South Kurils. The Chichibu-Okutama Mountains in central Japan (belonging to the warm temperate zone at lower elevations) house 230 species [39].

An illustrative comparison can be done with the adjacent areas that are relatively well-studied: the Paramushir Island liverwort flora (with a clearly hemiarctic vegetation) includes 85 species [40]. Attu Island, situated in a similar zone, counts 112 species [41]. However, these islands are located in a hemiarctic vegetation zone. Hokkaido Island, mentioned above, is much larger. Among smaller islands situated near (in similar climatic conditions with the South Kurils) is Rishiri Island, counting 92 species in a small-sized area of only 183 square km. The large hemiboreal flora of the southern part of Primorsky Territory (approximately 80,000 km^2^) includes approximately 150 species ([42], with our additions). When comparing some local floras in temperate Europe, the South Kurils are also ahead of the majority of districts. The list of liverworts of Auvergne [43] includes 189 species for land covering 13,796 square km. The Alpi Apuane counts 128 species [44] known on the land approximately 1000 square km in size. The Azores house 153 species on 2351 square km of land.

**Figure 1:**
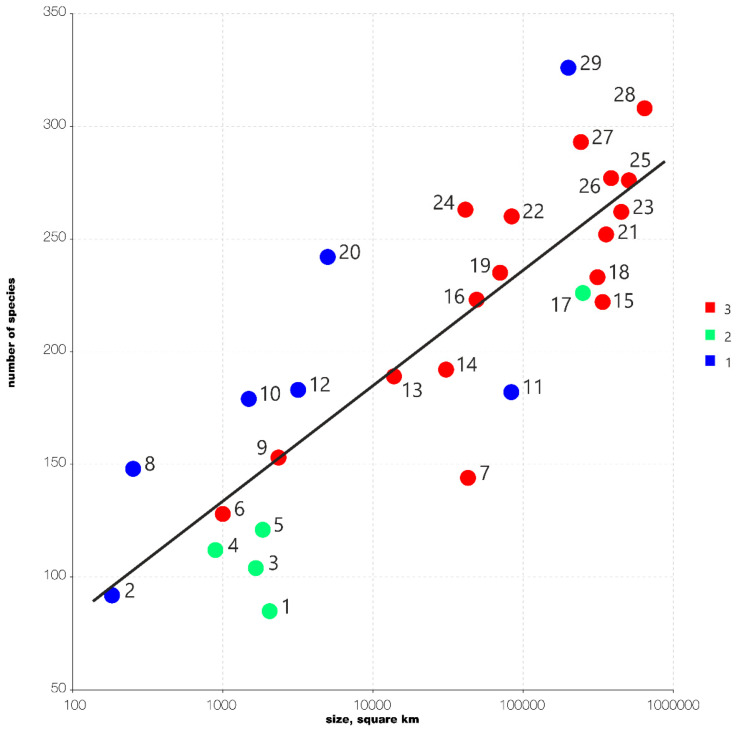
Species-area diagram based on data from Table 1. (**1**) Blue dots—hemiboreal and temperate floras in East Asia; (**2**) green dots—floras in hemiarctic Northeast Asia; (**3**) red dots—floras in Europe. Arrhenius SAR fitted for European floras.

Therefore, it was found that the flora of the South Kurils is richer than many larger places; the species richness of the South Kurils with 242 species in an area of <5000 square kilometers is comparable to the biodiversity of countries that are many times larger. We provide a graphical species-area diagram with species number plotted as a function of area (Figure 1). The Arrhenius SAR is fitted to the areas in Europe. The diagram is based on Table 1. As it easy to see, almost all hemiboreal and temperate floras in East Asia (blue colored dots) are above of the Arrhenius SAR (species-area relationships) fitted for Europe, whereas all hemiarctic sites in Northeast Asia (green colored dots) are below the Arrhenius SAR. This clearly highlights the diversity of the South Kurils versus other places in Eurasia. Meanwhile, the low position of Hokkaido Island (dot number 11 in Figure 1) evidently shows that its flora is strongly undercollected.

The comparison provided above is formalized in the provided diagram (Figure 1) and based entirely on Table 1.

The question arises: what causes such a high diversity of the liverwort flora in the South Kurils? Possible explanations may be (1) landscape diversity, including differentiation between islands, (2) position on the migration path from/to East Asia (cf. [45]), and (3) the island’s isolation effect.

The landscapes in the islands vary in lower elevations from gentle sloped plains to hilly plains (in fact, most of Shikotan Island is a hilly plain). At higher altitudes, the relief occasionally becomes similar to the alpine landscapes with steep slopes and narrow peaks: the most distinct examples are the near-top part of the Bogdan Khmelnitsky Volcano (Iturup) and in the immediate vicinity of Rurui Mt. (Kunashir). However, more often, the relief is intermediate between alpine-type and hilly, somewhat smoothed. The intensity of volcanic activity and age passed from the eruption events nearby (from modern to Miocene in the past) also affects the age of the substrata and the degree of their degradation, drainage abilities, and occupancy by vegetation. Towards the tops of the mountains, the amount of precipitation increases quite significantly (by 30%, exceeding that observed at lower altitude levels), whereas the temperatures decrease. In addition, the temperatures change along the latitudinal gradient (gently rising to the south). Landscape and climatic diversity suggest a diversity of community types ranging from broadleaved (including cool-temperate *Magnolia hypoleuca* Siebold and Zucc., *Fraxinus* L. spp., *Phellodendron sachalinense* (F. Schmidt) Sarg., *Kalopanax septemlobus* (Thunb.) Koidz.), dark coniferous (including cool-temperate *Taxus cuspidata* Siebold and Zucc.) forests, crooked forests, shrubby areas, *Sasa* impassable ‘meadows’, mountain tundra, and tundra-like communities on heavily wind-stressed slopes.

In addition to the diversity of landscapes and vegetation types, which increase the potential number of occurring plant species, the South Kurils are one of the inevitable links on the route of the most important floral exchange between Japan and Northeast Asia and even farther, with Pacific North America. In general, this phenomenon was formulated by Engler [46] and scrutinized by Takeda [47], Tatewaki [48], and others. Despite the active volcanism on the two largest islands of the South Kurils, which partially destroyed the flora (and, in this sense, impoverished its composition due to the ‘falling out’ of species that were not able to actively generate propagules), the South Kurils are a final destination for several species on the way to movement to the north and to the south. The most striking examples of migrants from the south are *Alobiellopsis parvifolia*, *Aneura maxima*, *Calypogeia japonica*, *Cololejeunea* spp., *Frullania* spp., *Hattorianthus erimonus*, *Nipponilejeunea pilifera,* and others, which do not pass the South Kurils, and the South Kurils are the northernmost distribution point in Pacific Asia. There are fewer migrants from the north that were ‘stopped’ in the South Kurils; among them are *Cephaloziella uncinata*, *C. elashista*, *Endogemma caesipticium*, and *Gymnomitrion concinnatum*; all those listed do not occur in Japan [38]. The macro-climate in the islands situated northward becomes much colder, and the North Kurils have typical hemiarctic vegetation, thus the conditions are probably not suitable for temperate taxa. Another reason for the existence of the area edge of southern taxa here are the wide oceanic straits between islands northward: the distance between Kunashir and Iturup Islands is ony 21 km, whereas the gap between Urup and Simushir Islands is 107 km, and from Simushr to Onekotan Island is 291 km, with only small islates between them that potentially make the probability of dispersal lower.

Finally, the third (particularly difficult to measure) factor promoting the high taxonomic diversity of liverworts in the South Kurils is the isolation effect in the islands. In this series, Shikotan Island stands out. This island is located away from the Great Kuril Chain, having no active volcanism evidence starting from the Miocene and is the final destination point of migration from the south in the Lesser Kuril Chain because there are no more islands immediately northwards of Shikotan. The presence of *Dasiphora fruticosa*, which is absent in Kunashir, and the absence of dwarf pine (*Pinus pumila* (Pall.) Regel), common on other large islands of the South (and North) Kurils, are widely known examples of Shikotan specificity [1]. The isolation from other islands of the South Kurils and Hokkaido is due to the high distance of Shikotan from the nearest island of the South Kurils (Kunashir), along with separation from Hokkaido Island through the long but flat Nemuro Peninsula (reducing the potential for migration of oro-boreal and arctomontane species), small-sized and flat Habomai Islets, and oceanic straits. This isolation might contribute to the conservation of a number of species that are currently not known on other islands of the South Kurils. There are 17 species known on Shikotan and not recorded in other South Kurils islands. Interestingly, six of them (*Cololejeunea subkodamae*, *Lejeunea flava*, *Lejeunea otiana*, *Nardia insecta*, *Pseudomoerckia blyttii*, *Riccia huebeneriana*, *Scapania mucronata*) are not known even in Hokkaido (the main potential ‘source’ of liverworts for Shikotan Island), and two more (*Moerckia flotoviana*, *Nardia insecta*) are not seen in Japan at all. However, considering the incomplete data on the diversity of liverworts in Hokkaido, at least some of the listed taxa may be expected there.

**Figure 2:**
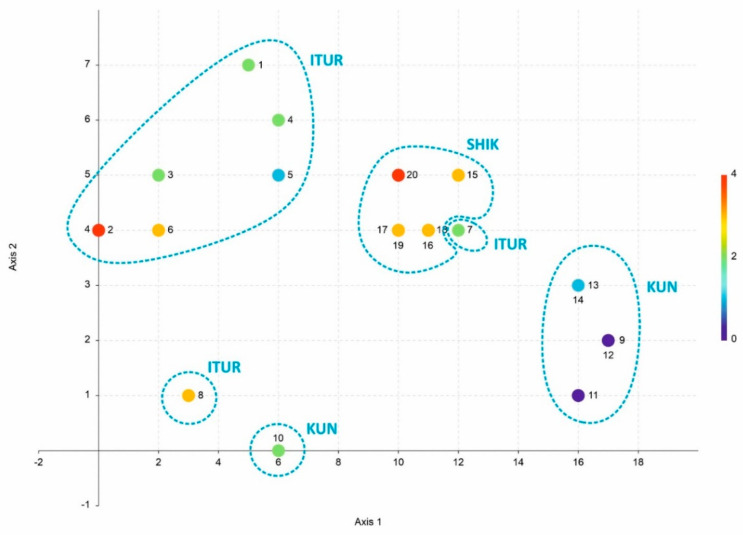
Results of bioclimates (Table S1) comparison by the DCA method.

A comparison of bioclimates following Table S1 by the DCA method is presented in Figure 2.

Prior to statistical comparison, it was assumed that the landscape and orographic position would be decisive for the relationship between climate data from different localities (conditionally speaking, all localities in the lower altitudinal belt could be united, whereas the localities from higher elevations should form a separate cluster). Indeed, the data on the average annual temperature (Table S1) seemed to confirm this assumption. However, when all available data were analysed together, it was found that the localities (with three exceptions) formed fairly clear clusters corresponding to the islands. Moreover, in Figure 2, the sequence of clusters corresponds to the island sequence in the direction of Iturup-Shikotan-Kunashir. The loosest cluster is formed by the climate data for localities on Iturup Island. In addition, two localities are separated from the group of all other Iturup localities. The climate in one of the highest elevations of the island (the crater of the Stockap Mountain (actually dormant volcano), point 8 in Figure 2) with a rather cold climate is left out. Point 10 is nearest to it and belongs to the atrio of Tyatya Volcano in Kunashir. Both localities are characterized by a humid cool climate with negative mean annual temperatures. It is interesting that the peak of Kamuy Mt. (1200 m a.s.l. in the very north of the South Kurils) is clearly localized with other Iturup local climates (completely merging with the climate of the Vetrovoy Isthmus, point 2 in Figure 2). Another point distanced from the rest of Iturup climate localities is point 7. This locality characterizes the climate of the isthmus to the Atsonopuri Volcano—one of the few places on Iturup Island where good coniferous forests of *Picea jezoensis* (Siebold & Zucc.) Carrière are developed. In this sense, the embedding of this locality into the Shikotan cluster is quite understandable (*Picea jezoensis* is the most common tree on Shikotan Island). These differences in the climatic conditions confirm that the differences observed in the floral composition of each island are not stochastic effects arising from undercollecting.

**Figure 3:**
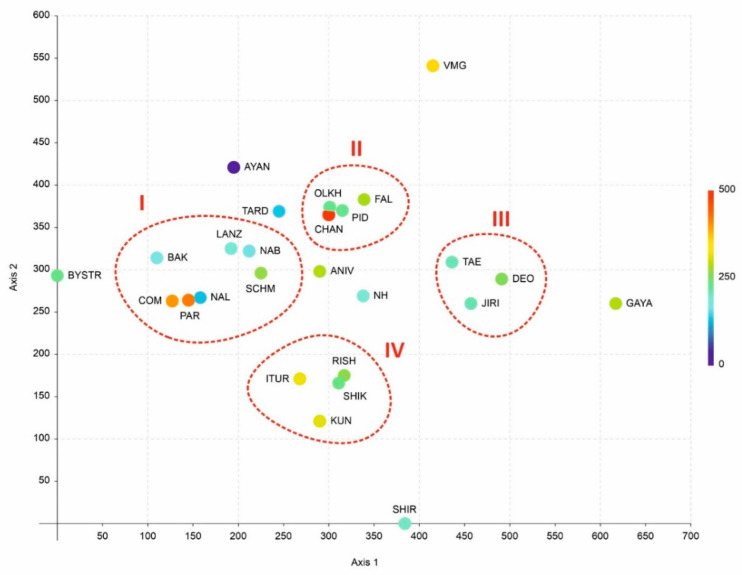
Comparison of the flora distribution in the DCA bubble chart (the third axis is the colour gradient from deep blue to deep red). Explanations on clusters are in the text and as following: I—hemiarctic suboceanic and oceanic floras, II—cool-temperate mainland floras (Manchurian floras), III—mountain floras of South Korea, IV—hemiboreal oceanic floras.

An analysis of the phytogeographic position of the small-sized floras (similar in size to the islands considered in the present account involved in the comparison) showed the results to be somewhat similar, but more statistically sound, in comparison with what was revealed in the previous study by Bakalin et al. [6]. The South Kurils cluster (IV) is indeed distanced from true East Asian (III) and circumboreal floras (I) (Figure 3).

This cluster (IV) also clearly includes the flora of Rishiri Island. Mountain flora with a full elevation gradient of vegetation (from temperate broadleaved forests to crooked forests and dark coniferous forests near the peaks) of the middle part of South Korea (III) are also combined into one clade. The southern Sikhote-Alin floras and Changbaishan also form one clade (II) (although it should be noted that the apparent proximity of Changbaishan Mt. flora to Olkhovaya Mt. flora in the diagram is just apparent, since these floras are quite far away along the third axis, shown by the colour gradient). The oceanic floras of the Kamchatka Peninsula and Sakhalin Island form another distinct cluster (I). Eight local flora are not included in any of the clusters. The reasons for this exclusion are insufficient knowledge (a small number of species, which makes the analysis unreliable), regional (including climatic) features, and incompleteness of the spectrum of communities along the altitudinal profile. Thus, the flora of Bystrinsky Nature Park is quite rich but developed in a subcontinental climate, which leads to differences in its structure from suboceanic floras. The flora of Ayan surroundings belongs to the hemiarctic floras of the Sea of Okhotsk coast, with a subcontinental climate and dry winters. Tardoki-Yani Range flora occupy an intermediate position between the floras of southern Sikhote-Alin and the oceanic floras of Kamchatka and Sakhalin. The latter is understandable considering its intermediate position between these clusters, both geographically and climatically. The flora of Shirakami Mt., located in the north of Honshu Island, belongs to the class of more southern flora, and it is also clearly insufficiently studied. The flora of Gaya-san does not include a sufficient number of species of the upper belts. In general, the abscissa (horizontal) axis quite clearly coincides with the latitudinal position of the floras, with one striking exception regarding the position of Shirakami.

The liverwort flora of the South Kurils is unique among other regional floras in Russia. Only twenty species are known in Russia from the South Kurils (*Alobiellopsis parvifolia*, *Calypogeia arguta*, *Calypogeia japonica*, *Calypogeia subalpina*, *Frullania usamiensis*, *Lejeunea flava*, *Lejeunea otiana*, *Lophozia obscura*, *Marsupella alata*, *Marsupella disticha*, *Microlejeunea punctiformis*, *Nipponolejeunea pilifera*, *Plectocolea hattoriana*, *Plectocolea rigidula*, *Riccardia subalpina*, *Riccardia vitrea*, *Solenostoma bilobum*, *Solenostoma emarginatum*, *Solenostoma hokkaidense*, *Wiesnerella denudata*). All of them, with the exception of *Wiesnerella denudata* (having a broader distribution, although with an area core in East and Southeast Asia), are chiefly East Asian in distribution.

## 3. Materials and Methods

To analyze the specifity and the position of the liverwort flora of the South Kurils among floras of Northeast Asia, we worked in three directions: (1) to compile the checklist of the South Kuries using available herbarium collections and literature data, (2) to compile the database on liverwort distribution within selected local floras in Northerast and cool-temperate East Asia, and (3) to collect available data for the comparison of taxonomic richness with some European countries and natural units (such as Alpi Apuane and Auvergne). More details are discussed below.

The available herbarium specimens of taxa whose distribution in South Kurils might be questioned in light of the currently accepted distribution concepts were revised. A suite of the new materials was collected in 2015 (Iturup Island), 2018 (Kunashir Island), and 2020 (Shikotan Island) and subsequently identified. The field expedition of 2015 to Iturup Island was remarkable since a group of bryologists (both moss and liverwort specialists) from various Russian institutes have visited Iturup Island, where they carried out a study mainly in the northern part of the island, which previously remained completely unexplored. Based on these collections, some newly found liverwort species on the island were mentioned by Bakalin et al. [6], but their locations were not provided in the paper. Several new distribution data were published in some taxonomic papers, including those on Ricciaceae [29], Jubulaceae [49], Lejeuneaceae [33], Radulaceae [28], Frullaniaceae [18] and several others cited in the checklist.

**Figure 4:**
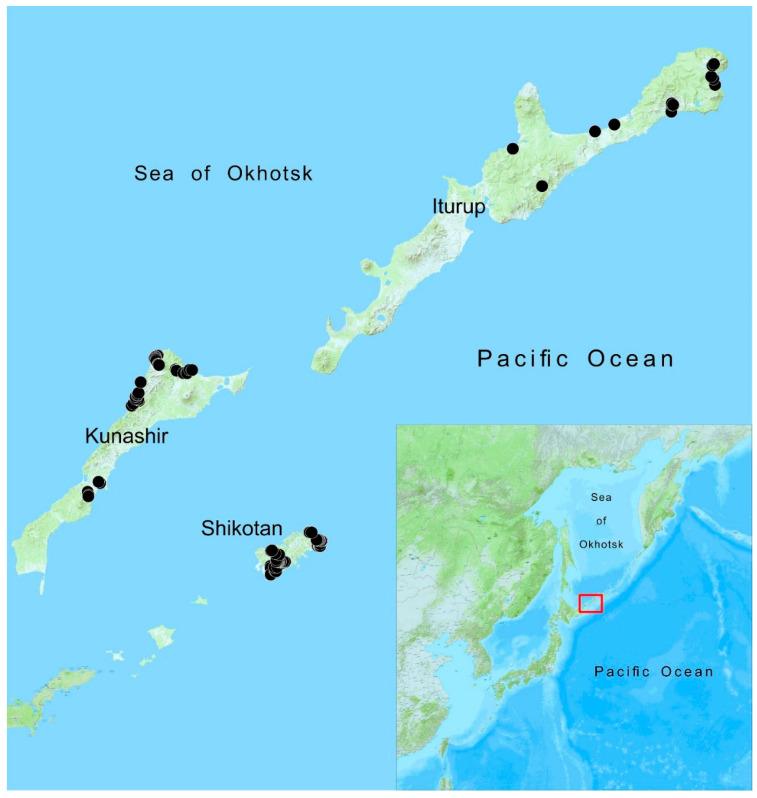
Collection localities by author team in the South Kurils in 2015–2020 corresponding to Table 3. Map with marked locality numbers is provided in Figure S1.

Data on the area and latitudinal characteristics of the islands are shown in Table 2, and the places of liverwort collection in the last ten years are shown on the map (Figure 4) and Table 3. The Habomai Islands were not studied, but data on them are provided for comparison in Table 2. The total area of the South Kurils is 5018 km^2^ (4966 km^2^ without Habomai). It should be noted that, in contrast to our previous work [6], we did not classify the islands northwards of Iturup as the South Kurils. This was done based on (1) the lack of at least minimally appropriate data on liverworts of these islands and (2) the lack of new information on these islands, which would enrich our work and would allow us to discuss the position of these islands from a phytogeographical point of view.

General data on the climate of the South Kurils were provided by Bakalin et al. [4]. All climate data provided before (l.c.) are based on data from weather stations located in large settlements, at low altitudes, and in open areas. Although the general trends are universal across the South Kurils, the climate is oceanic, cool, with the coldest month being February, with high snow cover (up to 2.5 m) and cool, humid summers. However, they hardly reflect the existing climate under which liverworts survive. Since it is obvious that the meso-relief elements and the elevation above sea level should influence the local climate features, we compiled a list of localities (some of those are the same as the collecting localities) for which we obtained data on 19 bioclimates (https://www.worldclim.org/, accessed on 24 February 2020). The bioclimate data are provided in Table S1 using the following abbreviations: BIO1 = Annual Mean Temperature; BIO2 = Mean Diurnal Range (Mean of monthly (max temp—min temp)); BIO3 = Isothermality (BIO2/BIO7) (×100); BIO4 = Temperature Seasonality (standard deviation ×100); BIO5 = Max Temperature of Warmest Month; BIO6 = Min Temperature of Coldest Month; BIO7 = Temperature Annual Range (BIO5-BIO6); BIO8 = Mean Temperature of Wettest Quarter; BIO9 = Mean Temperature of Driest Quarter; BIO10 = Mean Temperature of Warmest Quarter; BIO11 = Mean Temperature of Coldest Quarter; BIO12 = Annual Precipitation; BIO13 = Precipitation of Wettest Month; BIO14 = Precipitation of Driest Month; BIO15 = Precipitation Seasonality (Coefficient of Variation); BIO16 = Precipitation of Wettest Quarter; BIO17 = Precipitation of Driest Quarter; BIO18 = Precipitation of Warmest Quarter; BIO19 = Precipitation of Coldest Quarter.

Since the highest precision with which the bioclimates may be measured is 30″, this parameter clearly illustrates mesoclimatic conditions, not microclimatic conditions. Therefore, bioclimate data can be used only with certain reservations in regard to analysing bryophytes commonly growing in microniches. However, as a general pattern descriptor, the bioclimates work and correlate quite well with, for instance, the fact that the vast majority of arctomontane species grow in the tundra belt and do not occur in coniferous-broadleaved forests despite the great variation in microclimates.

Table S1 shows that the average annual temperature varies from slightly negative values in the apical parts of the mountains to +5 °C at lower altitudes. The total amount of precipitation varies from 1000 to 1400 mm, and it clearly increases with elevation above sea level and (to a lesser extent) in the direction from west to east. When comparing pairs of bioclimates, BIO16, 17 and BIO18, 19, it is obvious that the coldest quarter is also the driest (for Kuril bryophytes, this is not as important since the snow cover still covers most of the habitats). At the same time, the wettest quarter is not the warmest, although they distinctly overlap one another (the first shifts closer to the beginning of summer).

To describe in terms of statistics whether the climate characteristics are different from one island to another and to identify the value changes of the climate with elevation, we used the DCA method, the matrix for which is shown in Table S1. This method was previously used in a similar situation in the work with North Vietnam liverwort distribution [50].

**Figure 5:**
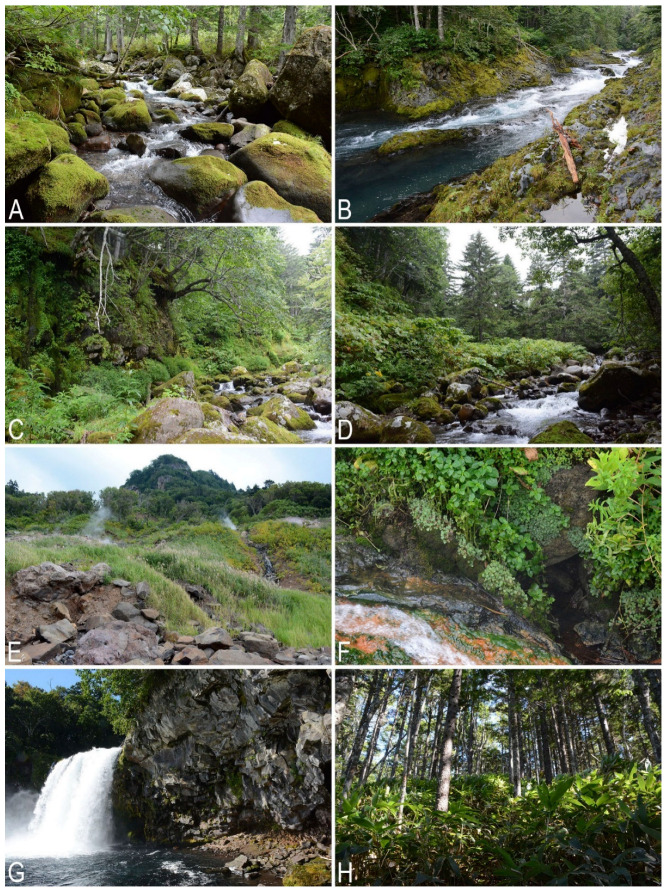
Liverwort habitats on Kunashir Island: (**A**,**C**,**D**) Gornaya River valley, northern part of the island, stream valeys in lower altitudinal belt; (**B**) Ptichiya River valley, northern part of the island, river valley; (**E**) coastal slope, Neskuchenskiye Hot Springs area, northern part of the island; (**F**) *Marchantia paleacea* along thermal stream, surrondings of Dal’ny Stream mouth, northern part of the island; (**G**) Ptichiya River mouth, northern part of the island; (**H**) *Abies nephrolepis* (Trautv. ex Maxim.) Maxim. forest with *Sasa* understory, middle part of the island (Photo by K.G. Klimova, 2018).

**Figure 6:**
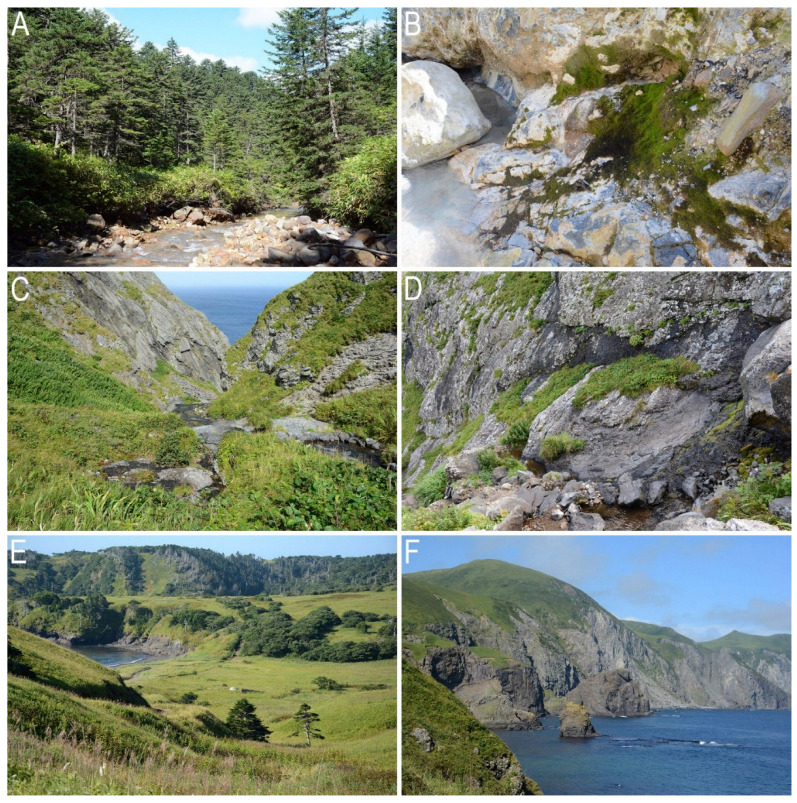
Liverwort habitats on Kunashir and Shikotan Islands: (**A**) *Abies nephrolepis* forest in Kislaya River valley, foot of Mendeleeva Volcano, Kunashir Island Middle part; (**B**) *Metasolenostoma orientale* on a bank of Kislaya River, NE-facing slope of Mendeleeva Volcano, middle part of Kunashir Island. (Photo by K.G. Klimova, 2018); (**C**) *Scapania gigantea* habitat, stream to sea in coastal cliffs, Shikotan Island; (**D**) sea-facing rocks—one of the habitat *of Gymnomitrion faurieanum* and *Radula brunnea*, Shikotan Island; (**E**) Krab Cape surroundings, Shikotan Island; (**F**) northeast rocky coast of the island, northward of Krab Cape, Shikotan Island (Photo by K.G. Klimova, 2020).

**Figure 7:**
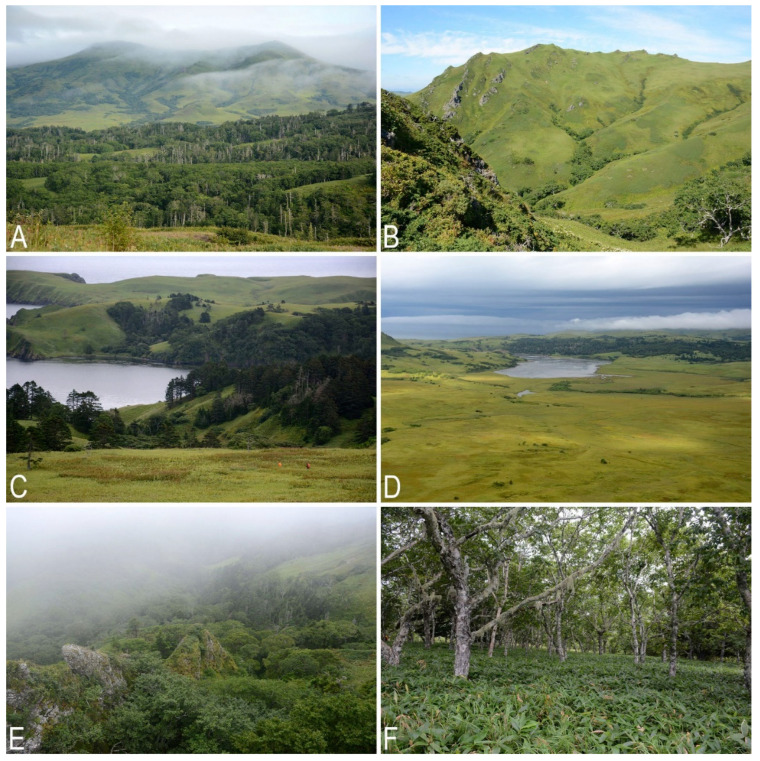
Liverwort habitats on Shikotan Island, southern part of the island: (**A**) Tomari Mt., view from the northwest, with *Picea-Abies* forests near the foot; (**B**) tundroid community (*Sasa, Eubotryoides, Empetrum*, grasses, etc.) formed under severe wind condition, with low (less 50 cm in height) clumps of *Juniperus sargentii*, inner part of the Notoro Mt.; (**C**) *Picea jezoensis* forest in Tserkovnaya Bay area; (**D**) hummocky swamp complex with *Carex, Phragmites, Dasiphora, Sphagnum,* and green mosses southeastward of Delphin Bay; (**E**) extensive rocky outcrops on steep slope covered by *Alnus* forest with some *Abies-Picea* clumps and tundroid communities (*Sasa, Eubotryoides, Empetrum,* grasses, etc.), northern spur of Ploskaya Mt.; (**F**) *Betula ermanii* forest with *Sasa* understory (Photo by K.G. Klimova, 2020).

The South Kurils flora was variably treated in various phytogeographic classifications, although almost all classifications referred to it as the East Asian floristic region. Various views on this issue were discussed by Bakalin [51]. Contrary to floristic regionalization, the vegetation of the lower altitudinal belts was recognized as hemiboreal starting from Ahti et al. [52] and is now widely accepted. Although it is worth mentioning, in the large-scale altitudinal variation (with mountain tundra vegetation in upper reaches) across the South Kurils, it is possible to postulate the vegetation as hemiboreal only in a very conditional sense. The main features of vegetation and landscapes were described in our previous work [4] and do not need to be repeated here. However, we provide the most characteristic types of habitats in Figure 5, Figure 6 and Figure 7.

As noted above, the collegial fieldwork in the northern part of Iturup Island carried out in 2015 did not result in a taxonomic checklist (although several species were mentioned in the paper text). However, the attempt to understand the phytogeographic position of Iturup and adjacent islands was made based on data on both moss and liverwort distributions (as far as it was available at that moment) in North Pacific Asia. It was suggested that the bryophyte flora of South Kurils belongs to its own phytogeographic province, which occupies an intermediate position between the East Asian and circumboreal floristic regions but does not definitely belong to either region. The intermediate nature of the liverwort flora of the South Kurils (between the Circumboreal and East Asian floristic regions) was shown even earlier [45].

Since 2009, the data on the taxonomic diversity of the South Kuril Islands have been significantly supplemented in the course of conducted work. The taxonomic lists of each island were used as a single unit in the analysis. Fortunately, for comparison purposes, progress was also achieved in the Korean Peninsula, where three more national park liverwort floras have been published since 2009. Each national park in Korea is somewhat comparable by the area with other floras involved in the analysis. The latter made it possible to enrich the matrix with new data, making the comparison more reliable. The compared floras are listed in Table 4. The floras located far to the north from South Kurils (areas in Northeast Asia with continental to ultracontinental climates) were removed from the matrix used before, which made it possible to avoid the visual effect of “sticking together” of taxonomically distant floras in hemiboreal and cool temperate amphi-Pacific Asia. The compiled matrix was analysed by the DCA method, as was also done in the previous work on liverworts from the vicinity of the Ayan Settlement [53] and in the abovementioned work on Northern Iturup [6] and North Vietnam [50]. In total, 475 taxa were involved in the analysis.

## 4. Conclusions

The isolation effect, combined with landscape diversity and position on the migration route from East to Northeast Asia, led to the formation and preservation of the richest district liverwort hemiboreal flora in Eurasia. There are no floras equal in diversity and of similar sizes in Europe. In contrast, in Pacific Asia, the highest taxonomic diversity per small-sized area is reached south of Kyushu, where on the small island of Yakushima, only 505 km^2^ in size, 310 species of liverworts and hornworts are known [75]. This is, however, an absolutely incomparable case because the flora of Yakushima Island in lower elevation belts is clearly subtropical.

It is difficult to imagine how much more the number of known taxa in the South Kurils will increase in future studies; however, given that the southern tip of Iturup Island has not yet been studied, there are no or only fragmentary data on a number of areas of Kunashir Island (especially in its middle part), and the liverwort systematic progress means the description of a coupes of new taxa, several new findings are highly probable. Considering the high taxonomic diversity of the liverworts, it is likely that conservation measures are needed to preserve the taxonomic diversity in this pristine corner of hemiboreal insular amphi-Pacific Asia.

## Figures and Tables

**Table 1:** The data on taxonomic diversity and area size in selected floras used for graphic diagram presented in Figure 1. The data on diversity are from [36,37,38,41,42,43,44] and the present paper. The data on the area are from https://en.wikipedia.org/wiki/Main_Page, (accessed on 19 August 2022).

Code in the Figure 1	Land Name	Size, Square km (Sometimes Approximate)	Number of Species	Color in Figure 1
1	Paramushir Island	2053	85	green
2	Rishiri Island	183	92	blue
3	Bering Island	1660	104	green
4	Attu Island	893	112	green
5	Commander Archipelago	1846	121	green
6	Alpi Apuane (natural region in Italy)	1000	128	red
7	Denmark	42,933	144	red
8	Shikotan Island	253	148	blue
9	Azores (the region of Portugal)	2351	153	red
10	Kunashir Island	1490	179	blue
11	Hokkaido	83,424	182	blue
12	Iturup Island	3175	183	blue
13	Auvergne Mts. (two departments in France)	13,796	189	red
14	Belgium	30,689	192	red
15	Finland	338,455	222	red
16	Slovakia	49,035	223	red
17	Kamchatka Peninsula	250,000	226	green
18	Poland	312,696	233	red
19	Ireland	70,273	235	red
20	South Kurils total	5000	242	blue
21	Germany	357,022	252	red
22	Austria	83,879	260	red
23	Sweden	450,295	262	red
24	Switzerland	41,285	263	red
25	Spain	505,990	276	red
26	Norway	385,207	277	red
27	Great Britain	242,495	293	red
28	France	643,801	308	red
29	Korean Peninsula	200,000	326	blue

**Table 2:** The physiographical data on islands under consideration.

Island	Area, km^2^	Coordinates	Length, km
Iturup	3175	45°33′–44°25′ N 148°53′–146°51′ E	199.2
Kunashir	1490	44°30′–43°39′ N 146°35′–145°24′ E	123.6
Shikotan	253	43°53′–43°41′ N 146°55′–146°35′ E	26.9
Habomai (as archipelago)	100	43°39′–43°21′ N 146°26′–145°53′ E	51.7

**Table 3:** The collection localities.

Locality Number	Geographic Description	Coordinates	Elevation above Sea Level, m	Collectors	Collection Date
**Iturup Island**					
**K-69**	Northern part of the island, Tsirk Bay, Tsirk River lower course	45°20.033′ N 148°37.067′ E	13	V.A. Bakalin	9 September 2015
**K-70**	Northern part of the island, Tsirk Bay, Tsirk River middle course	45°21.75′ N 148°37.217′ E	23	V.A. Bakalin	10 September 2015
**K-71**	Northern part of the island, Tsirk Bay, Tsirk River lower course	45°20.033′ N 148°37.067′ E	13	V.A. Bakalin	11 September 2015
**K-72**	Northern part of the island, Tsirk Bay, Tsirk River lower course	45°21′26” N 148°37′11” E	20	V.A. Bakalin	12 September 2015
**K-73**	Northern part of the island, Tsirk Bay, coastal cliffs	45°21.433′ N 148°37.583′ E	20	V.A. Bakalin	13 September 2015
**K-74**	Northern part of the island, Medvezhiya Bay	45°25.55′ N 148°49.867′ E	15	V.A. Bakalin	14 September 2015
**K-75**	Northern part of the island, Medvezhiya Bay, Medvezhiya River lower course	45°27.267′ N 148°48.767′ E	26	V.A. Bakalin	15 September 2015
**K-76**	Northern part of the island, Medvezhiya Bay, Medvezhiya River middle course	45°29.45′ N 148°49.1′ E	530	V.A. Bakalin	16 September 2015
**K-77**	Northern part of the island, Medvezhiya Bay, Nival’nyy Stream Upper course	45°29.767′ N 148°49.517′ E	750	V.A. Bakalin	16 September 2015
**K-78**	Northern part of the island, Medvezhiya Bay, Nival’nyy Stream Lower course	45°26.767′ N 148°49.383′ E	350	V.A. Bakalin	17 September 2015
**K-79**	Middle part of the island, Goryachaya River upper course	45°04.667′ N 147°59.217′ E	200	V.A. Bakalin	19 September 2015
**K-80**	Middle part of the island, Parusnaya Bay	45°11.467′ N 148°20.683′ E	5–30	V.A. Bakalin	20 September 2015
**K-81**	Middle part of the island, Belyye Skaly cliffs	45°15.967′ N 148°14.733′ E	5–15	V.A. Bakalin	20 September 2015
**K-82**	Middle part of the island, Rybaki Settlement area	45°12.383′ N 147°50.65′ E	5–15	V.A. Bakalin	21 September 2015
**Kunashir Island**					
**K-20**	Northern part of the island, Severyanka River valley lower course	44°20.383′ N 146°00.267′ E	74	V.A. Bakalin and K.G. Klimova	24 August 2018
**K-21**	Northern part of the island, Severyanka River valley lower course	44°20.267′ N 146°00.95′ E	18	V.A. Bakalin and K.G. Klimova	24 August 2018
**K-22**	Northern part of the island, Severyanka River valley lower course	44°20.133′ N 146°01.1′ E	27	V.A. Bakalin and K.G. Klimova	24 August 2018
**K-23**	Northern part of the island, Severyanka River valley lower course, small stream near river mouth	44°19.367′ N 145°59.567′ E	25	V.A. Bakalin and K.G. Klimova	25 August 2018
**K-24**	Northern part of the island, Severyanka River valley lower course, area along sea coast	44°18.933′ N 145°59.083′ E	13	V.A. Bakalin and K.G. Klimova	25 August 2018
**K-25**	Northern part of the island, Severyanka River valley lower course, area along sea coast	44°19.367′ N 145°59.567′ E	13	V.A. Bakalin and K.G. Klimova	25 August 2018
**K-26**	Northern part of the island, Severyanka River valley lower course, area along sea coast	44°18.933′ N 145°59.083′ E	61	V.A. Bakalin and K.G. Klimova	25 August 2018
**K-27**	Northern part of the island, Severyanka River valley lower course	44°19.967′ N 146°01.25′ E	22	V.A. Bakalin and K.G. Klimova	26 August 2018
**K-28**	Northern part of the island, Zolotaya River (large sulphureous stream) valley lower course, area near river mouth	44°20.833′ N 146°00.2′ E	14	V.A. Bakalin and K.G. Klimova	27 August 2018
**K-29**	Northern part of the island, Zolotaya River (large sulphurous stream) valley lower course	44°20.833′ N 146°00.2′ E	14	V.A. Bakalin and K.G. Klimova	27 August 2018
**K-30**	Northern part of the island, Zolotaya River (large sulphurous stream) valley lower course	44°21.2′ N 146°00.617′ E	30	V.A. Bakalin and K.G. Klimova	27 August 2018
**K-31**	Northern part of the island, Zolotaya River (large sulphurous stream) valley lower course	44°21.25′ N 146°00.833′ E	36	V.A. Bakalin and K.G. Klimova	27 August 2018
**K-32**	Northern part of the island, Zolotaya River (large sulphurous stream) valley lower course	44°21.567′ N 146°01.167′ E	49	V.A. Bakalin ous K.G. Klimova	27 August 2018
**K-33**	Northern part of the island, Gornaya River valley, confluence of two small rivulets in the middle course of Gornaya River	44°26.267′ N 146°12.2′ E	213	V.A. Bakalin and K.G. Klimova	30 August 2018
**K-34**	Northern part of the island, Gornaya River valley, middle course	44°26.367′ N 146°12.167′ E	220	V.A. Bakalin and K.G. Klimova	30 August 2018
**K-35**	Northern part of the island, Gornaya River valley, middle course, narrow valley	44°26.55′ N 146°11.933′ E	280	V.A. Bakalin and K.G. Klimova	30 August 2018
**K-36**	Northern part of the island, Gornaya River valley, middle course, narrow valley	44°26.55′ N 146°11.933′ E	280	E.E. Kozlovsky	30 August 2018
**K-37**	Northern part of the island, Gornaya River valley, lower course, narrow valley	44°25.783′ N 146°14.2′ E	105	V.A. Bakalin and K.G. Klimova	31 August 2018
**K-38**	Northern part of the island, Ptichiya River lower course	44°25.75′ N 146°15.283′ E	30	V.A. Bakalin and K.G. Klimova	1 September 2018
**K-39**	Northern part of the island, Ptichiya River lower course, forest at the ridge near Nelyudimyy Cape	44°25.95′ N 146°15.15′ E	162	V.A. Bakalin and K.G. Klimova	1 September 2018
**K-40**	Northern part of the island, Ptichiya River lower course, area near waterfall at the river mouth	44°26.417′ N 146°16.7′ E	3-27	V.A. Bakalin and K.G. Klimova	2 September 2018
**K-41**	Northern part of the island, Ptichiya River lower course	44°26.45′ N 146°16′ E	90	V.A. Bakalin and K.G. Klimova	2 September 2018
**K-42**	Northern part of the island, Dal’niy Stream lower course, just above the first waterfall	44°28.833′ N 146°05.733′ E	132	V.A. Bakalin and K.G. Klimova	4 September 2018
**K-43**	Northern part of the island, Dal’niy Stream lower course, just below the third waterfall	44°28.75′ N 146°05.967′ E	178	V.A. Bakalin and K.G. Klimova	4 September 2018
**K-44**	Northern part of the island, Dal’niy Stream lower course, 300 m below main confluence with right tributary	44°28.633′ N 146°06.233′ E	260	V.A. Bakalin and K.G. Klimova	5 September 2018
**K-45**	Northern part of the island, Dal’niy Stream middle course	44°28.37′ N 146°06.257′ E	326	V.A. Bakalin and K.G. Klimova	6 September 2018
**K-46**	Northern part of the island, Dal’niy Stream middle course	44°27.653′ N 146°06.853′ E	630	V.A. Bakalin and K.G. Klimova	6 September 2018
**K-47**	Northern part of the island, Dal’niy Stream upper course	44°27.55′ N 146°06.905′ E	705	V.A. Bakalin and K.G. Klimova	6 September 2018
**K-48**	Northern part of the island, Dal’niy Stream upper course	44°27.477′ N 146°07.038′ E	774	V.A. Bakalin and K.G. Klimova	6 September 2018
**K-49**	Northern part of the island, Dal’niy Stream lower course, 300 m below main confluence with right tributary	44°28.633′ N 146°06.233′ E	260	V.A. Bakalin and K.G. Klimova	7 September 2018
**K-50**	Northern part of the island, Dal’niy Stream middle course	44°28.37′ N 146°06.257′ E	326	V.A. Bakalin and K.G. Klimova	7 September 2018
**K-51**	Northern part of the island, Dal’niy Stream middle course	44°27.653′ N 146°06.853′ E	630	V.A. Bakalin and K.G. Klimova	7 September 2018
**K-52**	Northern part of the island, coastal area near Dal’niy Stream mouth	44°29.125′ N 146°05.923′ E	15	V.A. Bakalin and K.G. Klimova	8 September 2018
**K-53**	Northern part of the island, coastal area near Dal’niy Stream mouth	44°29.205′ N 146°05.993′ E	6	V.A. Bakalin and K.G. Klimova	8 September 2018
**K-54**	Northern part of the island, Neskuchenskiye Hot Springs area	44°29.408′ N 146°06.577′ E	34	V.A. Bakalin and K.G. Klimova	8 September 2018
**K-55**	Northern part of the island, Neskuchenskiye Hot Springs area	44°29.47′ N 146°06.693′ E	50	V.A. Bakalin and K.G. Klimova	8 September 2018
**K-56**	Northern part of the island, Sea of Okhotsk coast, 3 km northward of Prasolova Cape	44°23.872′ N 146°01.712′ E	4	V.A. Bakalin and K.G. Klimova	9 September 2018
**K-57**	Middle part of the island, swampy massif at middle course of Serebryanka River	44°02.7′ N 145°49.8′ E	5	V.A. Bakalin and K.G. Klimova	10 September 2018
**K-58**	Middle part of the island, swampy Picea glehnii forest at middle course of Serebryanka River	44°02.95′ N 145°49.367′ E	7	V.A. Bakalin and K.G. Klimova	10 September 2018
**K-59**	Middle part of the island, Kislaya River valley lower course	44°00.838′ N 145°46.2′ E	40	V.A. Bakalin and K.G. Klimova	11 September 2018
**K-60**	Middle part of the island, Doktorsky Stream Valley upper course	43°59.988′ N 145°46.407′ E	84	V.A. Bakalin and K.G. Klimova	11 September 2018
**K-61**	Middle part of the island, Doktorsky Stream Valley upper course	44°00.047′ N 145°46.425′ E	73	V.A. Bakalin and K.G. Klimova	11 September 2018
**Shikotan Island**					
**K-38**	Notoro Mt. W-facing slope	43°46.331′ N 146°41.928′ E	250	V.A. Bakalin and K.G. Klimova	24 August 2020
**K-39**	Notoro Mt., area near the top, eastern slope, N-facing cliffs	43°46.311′ N 146°41.741′ E	320	V.A. Bakalin and K.G. Klimova	24 August 2020
**K-40**	Notoro Mt., area near the top, eastern spur, N-facing cliffs	43°46.57′ N 146°41.457′ E	350	V.A. Bakalin and K.G. Klimova	24 August 2020
**Shik-32**	Notoro Mt., the foot of the mountain	43°46.042′ N 146°42.108′ E	115	K.G. Klimova and V.A. Bakalin	24 August 2020
**Shik-33**	Notoro Mt., SE-facing slope	43°46.113′ N 146°42.008′ E	172	K.G. Klimova and V.A. Bakalin	24 August 2020
**Shik-34**	Notoro Mt., SE-facing slope	43°46.183′ N 146°41.927′ E	235	K.G. Klimova and V.A. Bakalin	24 August 2020
**Shik-35, Shik-36**	Notoro Mt., N-facing slope	43°46.175′ N 146°41.418′ E	323	K.G. Klimova and V.A. Bakalin	24 August 2020
**K-41**	Ploskaya Mt., northern spur	43°48.434′ N 146°39.847′ E	294	V.A. Bakalin and K.G. Klimova	25 August 2020
**Shik-37**	Ploskaya Mt., northern spur	43°48.432′ N 146°39.847′ E	295	K.G. Klimova and V.A. Bakalin	25 August 2020
**K-42, Shik-39**	Ploskaya Mt., eastern spur	43°48.474′ N 146°40.136′ E	276	V.A. Bakalin and K.G. Klimova, K.G. Klimova and V.A. Bakalin	25 August 2020
**K-43**	Ploskaya Mt., eastern spur	43°47.855′ N 146°41.044′ E	159	V.A. Bakalin and K.G. Klimova	25 August 2020
**Shik-38**	4 km southeastward of Ploskaya Mt.	43°47.587′ N 146°42.218′ E	75	K.G. Klimova and V.A. Bakalin	25 August 2020
**K-44**	Tomari Mt., eastern spur	43°45.843′ N 146°43.356′ E	230	V.A. Bakalin and K.G. Klimova	26 August 2020
**K-45**	Tomari Mt., eastern spur	43°46.069′ N 146°43.917′ E	351	V.A. Bakalin and K.G. Klimova	26 August 2020
**K-46**	Tomari Mt., area near the top, N-facing cliffs	43°46.019′ N 146°43.828′ E	300	V.A. Bakalin and K.G. Klimova	26 August 2020
**Shik-40**	Tomari Mt., NW-facing slope	43°46.069′ N 146°43.917′ E	351	K.G. Klimova and V.A. Bakalin	26 August 2020
**Shik-41**	Tomari Mt., NW-facing slope	43°46.13′ N 146°43.48′ E	217	K.G. Klimova and V.A. Bakalin	26 August 2021
**K-47**	Saddle between Notoro and Tomari Mts.	43°46.107′ N 146°42.626′ E	120	V.A. Bakalin and K.G. Klimova	26 August 2020
**K-48**	Tserkovnaya Bay surroundings	43°44.968′ N 146°40.843′ E	32	V.A. Bakalin and K.G. Klimova	27 August 2020
**K-49**	Tserkovnaya Bay surroundings	43°44.077′ N 146°41.328′ E	5	V.A. Bakalin and K.G. Klimova	27 August 2020
**Shik-42**	Small stream valley in Tserkovnaya Bay area	43°43.422′ N 146°39.78′ E	61	K.G. Klimova and V.A. Bakalin	28 August 2020
**K-50**	Delphin Bay surroundings	43°45.199′ N 146°39.703′ E	8	V.A. Bakalin and K.G. Klimova	29 August 2020
**Shik-43**	Southeastward of Delphin Bay	43°44.785′ N 146°39.76′ E	24	K.G. Klimova and V.A. Bakalin	29 August 2020
**K-51**	Tserkovnaya Bay	43°44.077′ N 146°41.328′ E	5	V.A. Bakalin and K.G. Klimova	29 August 2020
**K-52**	Tserkovnaya Bay	43°44.077′ N 146°41.328′ E	5	V.A. Bakalin and K.G. Klimova	30 August 2020
**Shik-44**	Vicinity of Tserkovnaya Bay	43°44.223′ N 146°41.038′ E	31	K.G. Klimova and V.A. Bakalin	30 August 2020
**Shik-45**	Vicinity of Tserkovnaya Bay	43°44.192′ N 146°41.088′ E	26	K.G. Klimova and V.A. Bakalin	30 August 2021
**Shik-46**	Valley of the river to Tserkovnaya Bay	43°44.975′ N 146°41.672′ E	16	K.G. Klimova and V.A. Bakalin	30 August 2022
**K-53, Shik-48**	Shikotan Mt., area near the top	43°52.301′ N 146°51.224′ E	386	V.A. Bakalin and K.G. Klimova, K.G. Klimova and V.A. Bakalin	31 August 2020
**Shik-47**	Shikotan Mt., area near the top	43°52.323′ N 146°51.045′ E	342	K.G. Klimova and V.A. Bakalin	31 August 2020
**K-54**	Kray Sveta Cape area	43°50.513′ N 146°54.626′ E	5	V.A. Bakalin and K.G. Klimova	1 September 2020
**Shik-50**	Kray Sveta Cape area	43°50.513′ N 146°54.508′ E	8	K.G. Klimova and V.A. Bakalin	1 September 2020
**K-55**	Kray Sveta Cape area, narrow valley of small stream northward of the cape	43°50.547′ N 146°53.925′ E	50	V.A. Bakalin and K.G. Klimova	1 September 2020
**Shik-51**	Kray Sveta Cape area, narrow valley of small stream westward of the cape	43°50.563′ N 146°54.002′ E	48	K.G. Klimova and V.A. Bakalin	1 September 2020
**K-56**	Krab Cape area	43°49.87′ N 146°54.199′ E	5	V.A. Bakalin and K.G. Klimova	1 September 2020
**Shik-49**	stream valley near small nameless bay southward of Krab Cape	43°49.867′ N 146°54.195′ E	7	K.G. Klimova and V.A. Bakalin	31 August 2020
**K-57**	Kray Sveta Cape	43°50.674′ N 146°53.464′ E	193	V.A. Bakalin and K.G. Klimova	2 September 2020
**K-58**	Unnamed mountain northward of Kray Sveta Cape	43°50.73′ N 146°53.212′ E	371	V.A. Bakalin and K.G. Klimova	2 September 2020
**Shik-53**	About 1 km westward of Kray Sveta Cape	43°50.688′ N 146°53.678′ E	108	K.G. Klimova and V.A. Bakalin	2 September 2020
**Shik-52**	About 1 km westward of Kray Sveta Cape	43°50.76′ N 146°53.693′ E	78	K.G. Klimova and V.A. Bakalin	2 September 2021
**K-59, Shik-54**	Small bays and capes southward of Krab Cape	43°49.61′ N 146°53.921′ E	14	V.A. Bakalin and K.G. Klimova, K.G. Klimova and V.A. Bakalin	3 September 2020
**K-60, Shik-55**	Small bays and capes southward of Krab Cape	43°49.533′ N 146°53.913′ E	34	V.A. Bakalin and K.G. Klimova, K.G. Klimova and V.A. Bakalin	3 September 2020
**K-61, Shik-56**	Small bays and capes southward of Krab Cape	43°49.415′ N 146°54.245′ E	25	V.A. Bakalin and K.G. Klimova, K.G. Klimova and V.A. Bakalin	3 September 2020
**K-62, Shik-57**	Small bays and capes southward of Krab Cape	43°49.456′ N 146°53.562′ E	71	V.A. Bakalin and K.G. Klimova, K.G. Klimova and V.A. Bakalin	3 September 2020
**K-63, Shik-58**	Small bays and capes southward of Krab Cape	43°49.5′ N 146°54.087′ E	12	V.A. Bakalin and K.G. Klimova, K.G. Klimova and V.A. Bakalin	3 September 2020
**K-64**	Small bays and capes southward of Krab Cape	43°49.867′ N 146°54.195′ E	7	V.A. Bakalin and K.G. Klimova	3 September 2020

**Table 4:** The list of local flora involved in the comparison.

No	Abbreviations of Floras	Explanation of the Abbreviation, Literature Sources	Approximate Coordinates
1	**ANIV**	Aniva Bay and Aniva Peninsula in Sakhalin Island [54]	46°30′ N 142°30′ E
2	**AYAN**	Ayan Settlement surroundings, Dzhugdzhur Range, Pribrezhnyi Range [53]	56°27′ N 138°12′ E
3	**BAK**	Bakening Volcano and adjacent mountains in East Kamchatka [6]	54°00′ N 158°00′ E
4	**BYSTR**	Bystrinsky Nature Park, Sredinnyi Range, Central Kamchatka [55]	56°00′ N 158°30′ E
5	**CHAN**	Changbaishan Mts. in north-east China [56,57]	42°00′ N 128°00′ E
6	**COM**	Commander Islands [58,59]	55°00′ N 166°00′ E
7	**DEO**	Deokgyu Mts., Mt. Deogyu National Park, southern part of Korean Peninsula [60]	36°00′ N 127°30′ E
8	**FAL**	Litovka Mt., Livadysky Range, southern part of Sikhote-Alin Mts. [61]	43°06′ N 132°47′ E
9	**GAYA**	Gayasan Mts., Gayasan Mountain National Park, southern part of Korean Peninsula [62]	35°48′ N 128°06′ E
10	**ITUR**	Iturup Island, Kuril Islands [4,6]	45°00′ N 149°00′ E
11	**JIRI**	Jirisan Mts, Jirisan National Park, southern part of Korean Peninsula [63]	35°20′ N 127°40′ E
12	**KUN**	Kunashir Island, Kuril Islands [4,6,64]	44°00′ N 146°00′ E
13	**LANZ**	Lanzhinskiye Mts. in North Okhotiya [65]	59°30′ N 143°30′ E
14	**NAB**	Nabilsky Range of Sakhalin [54]	51°00′ N 143°00′ E
15	**NAL**	Nalychevo Nature Park, Nalycheva River valley and adjacent volcanoes in East Kamchatka [66]	53°30′ N 159°00′ E
16	**NH**	Shiretoko, Nemuro Abashiri Peninsulas, Hokkaido Island [67]	44°00′ N 145°00′ E
17	**OLKH**	Olkhovaya Mt., Alekseevsky Range, southern part of Sikhote-Alin Mts. ([68], unpublished data)	43°21′ N 133°39′ E
18	**PAR**	Paramushir Island, Kuril Islands [40]	51°30′ N 156°00′ E
19	**PID**	Livadyskaya Mt., Livadysky Range, southern part of Sikhote-Alin Mts. ([69], unpublished data)	43º04′ N 132º41′ E
20	**RISH**	Rishiri Island, opposite to western cost of Hokkaido Island [70]	45°00′ N 141°00′ E
21	**SCHM**	Schmidt Peninsula in Sakhalin Island [54]	54°00′ N 142°30′ E
22	**SHIK**	Shikotan Island, Kuril Islands ([4], data from the present account)	43°30′ N 143°30′ E
23	**SHIR**	Shirakami Mt., Aomori Prefecture, Honshu Island [71]	40°30′ N 140°00′ E
24	**TAE**	Taebaeksan Mts., Taebaeksan Mountain National Park, southern part of Korean Peninsula [72]	37°06′ N 128°55′ E
25	**TARD**	Tardoki Yani Range, northern part of Sikhote-Alin Mts. [73]	48°53′ N 138°02′ E
26	**VMG**	East-Manchurian Mts. (Kedrovaya Pad’ Nature Reserve, mountains westward of Razdolnaya River valley, Sinyaya Mt. [74], unpublished data)	43°07′ N 131°28′ E

## Data Availability

Not applicable.

## Supplementary Materials

The following supporting information can be downloaded at: https://www.mdpi.com/article/10.3390/plants11172200/s1, Figure S1: Collection localities by author team in southern Kurils in 2015–2020 corresponding to Table 3 (with marked locality numbers); Table S1: Bioclimatic data involved into analisys.
