# Supplementary figures and images for: The Taxonomically Richest Liverwort Hemiboreal Flora in Eurasia Is in the South Kurils
Source: Plants (Basel). 2022 Aug 25;11(17):2200. doi: 10.3390/plants11172200 (PMC9460601; doi:10.3390/plants11172200)

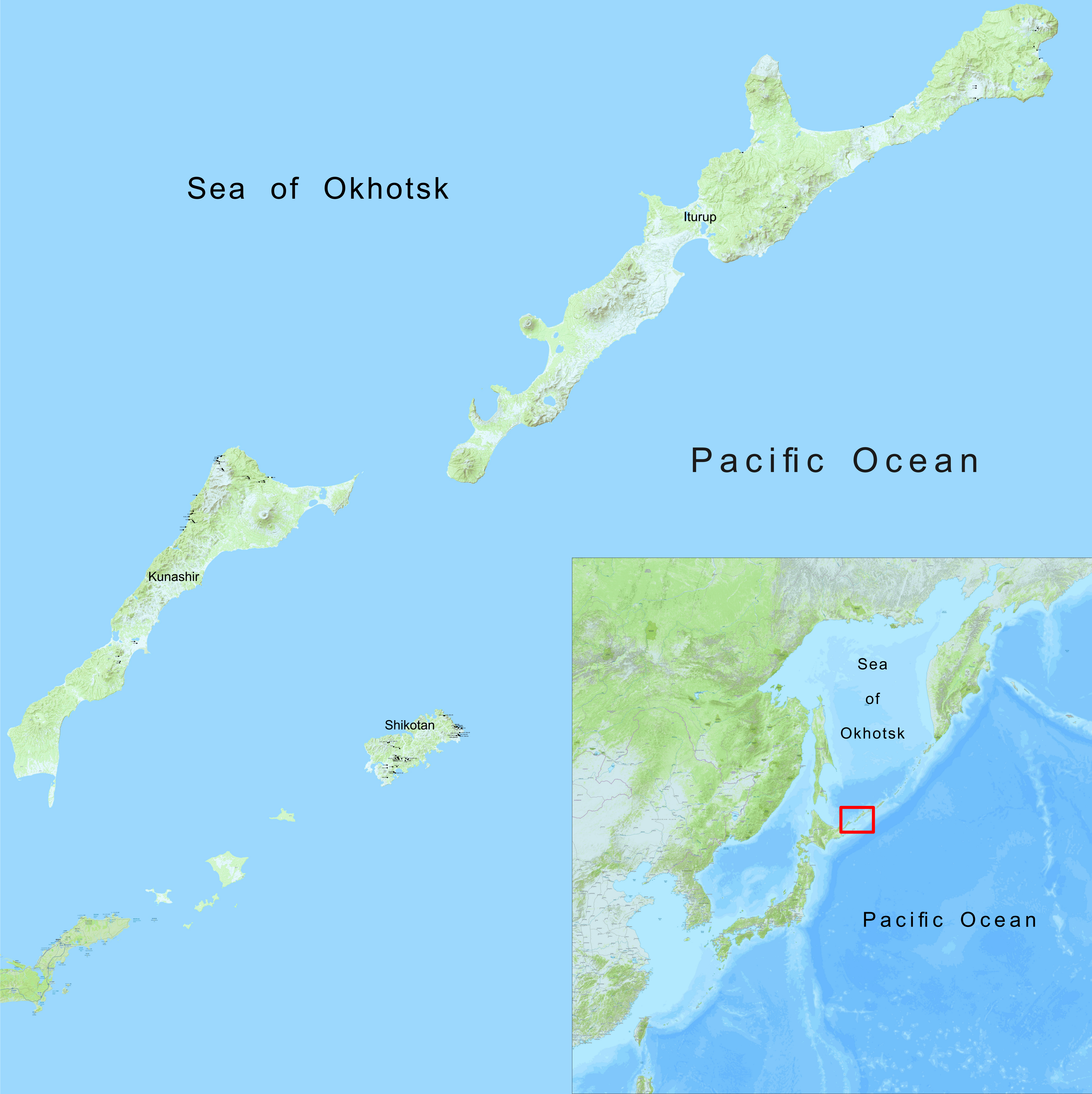

Supplement: Supplementary file 1 [file plants-11-02200-s001.zip › Figure S1.pdf]
